# Short Lifespans of Memory T-cells in Bone Marrow, Blood, and Lymph Nodes Suggest That T-cell Memory Is Maintained by Continuous Self-Renewal of Recirculating Cells

**DOI:** 10.3389/fimmu.2018.02054

**Published:** 2018-09-11

**Authors:** Mariona Baliu-Piqué, Myrddin W. Verheij, Julia Drylewicz, Lars Ravesloot, Rob J. de Boer, Ad Koets, Kiki Tesselaar, José A. M. Borghans

**Affiliations:** ^1^Laboratory of Translational Immunology, University Medical Center Utrecht, Utrecht, Netherlands; ^2^Department of Bacteriology and Epidemiology, Wageningen Bioveterinary Research, Lelystad, Netherlands; ^3^Department of Farm Animal Health, Faculty of Veterinary Medicine, Utrecht University, Utrecht, Netherlands; ^4^Theoretical Biology, Utrecht University, Utrecht, Netherlands

**Keywords:** bone marrow, memory T-cells, lymphocyte turnover, lifespan, stable isotope labeling, deuterium, mathematical modeling

## Abstract

Memory T-cells are essential to maintain long-term immunological memory. It is widely thought that the bone marrow (BM) plays an important role in the long-term maintenance of memory T-cells. There is controversy however on the longevity and recirculating kinetics of BM memory T-cells. While some have proposed that the BM is a reservoir for long-lived, non-circulating memory T-cells, it has also been suggested to be the preferential site for memory T-cell self-renewal. In this study, we used *in vivo* deuterium labeling in goats to simultaneously quantify the average turnover rates—and thereby expected lifespans—of memory T-cells from BM, blood and lymph nodes (LN). While the fraction of Ki-67 positive cells, a snapshot marker for recent cell division, was higher in memory T-cells from blood compared to BM and LN, *in vivo* deuterium labeling revealed no substantial differences in the expected lifespans of memory T-cells between these compartments. Our results support the view that the majority of memory T-cells in the BM are self-renewing as fast as those in the periphery, and are continuously recirculating between the blood, BM, and LN.

## Introduction

Immunological memory, the ability of the immune system to respond more quickly and strongly upon repeated antigen exposure, is the hallmark of the adaptive immune system. T-cell memory generated after the first antigen encounter can last for decades, and provides long-lasting immune protection (1, 2). It has convincingly been shown that T-cells with a memory phenotype in human blood renew quite often and are not maintained by a long cellular lifespan (3–11). It is important to realize, however, that most insights into memory T-cell maintenance in humans have been based on cells from peripheral blood. At any given moment in time only a very small fraction of the total body lymphocyte pool is present in the blood (12, 13), whereas the vast majority of memory T-cells are located in lymphoid and non-lymphoid tissues (14). This raises the question whether T-cell lifespan estimates based on cells from peripheral blood are also representative for T-cells located in tissues.

Physiological T-cell niches are an important factor in the maintenance of T-cell memory (15, 16). The bone marrow (BM) has recently attracted a lot of attention as a reservoir for memory T-cells (17–20). Although it is well established that memory T-cells are abundantly present in the BM, preferentially home there following infection (19, 21), and are able to expand in the BM following antigen re-challenge (22), the exact role of BM in the maintenance of T-cell memory is less clear (16, 23). BM has been shown to be a niche for memory T-cells that rest in terms of proliferation, transcription, and migration (18, 24). Hence, BM has been proposed as the place where memory T-cells with long lifespans reside. Other studies have suggested however that memory T-cells in BM are more actively proliferating than those in lymph nodes (LN) (17, 25), suggesting that BM provides the appropriate environment for memory T-cells to self-renew. In an attempt to reconcile the conflicting literature, it has been proposed that BM might provide two distinct niches for recirculating memory T-cells, one which supports cycling of memory T-cells, and another that provides a niche for quiescent memory T-cells (16, 26).

Studies addressing the dynamics of BM memory T-cells have used different models and techniques. In mouse studies, both kinetic markers, such as bromodeoxyuridine (BrdU) and carboxyfluorescein diacetate succinimidyl ester (CFSE) labeling (17, 25, 27), and static markers, such as Ki-67 expression (19, 24), have been used to determine the proliferative status of BM memory T-cells. Dynamic markers provide rich information on the division history of the cells, but BrdU labeling has been linked to cellular toxicity (24, 28) and CFSE labeling requires *ex vivo* cell manipulation, which may interfere with cell homeostasis. A static marker like Ki-67 describes the division status of a cell at a given moment and location, but provides no information about cellular lifespans, and does not take into account that a cell may have proliferated previously, or elsewhere. In human studies, only static markers have been used to assess memory T-cell proliferation in organs other than blood (18). Another point to consider is that in mouse experiments, cell dynamics in BM have typically been compared to those in lymphoid organs, while human studies have based their comparisons on blood-derived cells. The debate in the literature together with the array of different approaches used to estimate the lifespan of BM memory T-cells highlights the difficulty of assessing how memory T-cell populations are maintained, in particular those located outside the blood.

In this study, we simultaneously quantified the dynamics of memory CD4^+^ and CD8^+^ T-cells in BM, blood, and lymphoid organs using *in vivo* stable isotope labeling, the state of the art technique to study lymphocyte dynamics *in vivo*. One of the great advantages of this technique is that the turnover of a cell population is traced regardless of time and space, allowing us to reliably follow the division history of a population. In addition, *in vivo* deuterium labeling is non-toxic and does not require *ex vivo* cell manipulation, enabling the study of an unperturbed system. To simultaneously quantify the lifespans of memory CD4^+^ and CD8^+^ T-cells in blood, BM and lymphoid organs we made use of the goat as animal model, taking advantage of its relatively large size to obtain enough T-lymphocytes from paired samples of blood, BM, and LNs.

## Materials and methods

### Goats

Female adult goats (*N* = 34) were purchased from commercial farms and housed at Wageningen Bioveterinary Research, Lelystad, The Netherlands. Additional one-off surplus material from single blood samples taken for mandatory routine diagnostic tests were obtained from 8 adult female goats housed at the Department of Farm Animal Health, Faculty of Veterinary Medicine of the Utrecht University were used for IFN-ɤ ELISA assay.

### Ethics

This study was carried out in accordance with national regulations on animal experimentation. The protocol was approved by the animal experiment commissions of Wageningen Bioveterinary Research (permit number AVD401002016580).

### *In vivo* stable isotope labeling

Deuterated water (^2^H_2_O) (99.8%; Cambridge Isotope Laboratories) was diluted to 4% in drinking water and administered *ad libitum* for 28 days. To determine deuterium enrichment in the body water, heparin plasma was collected during the up- and down-labeling phase, and was frozen and stored at −20°C until analysis.

### Sampling and cell preparation

Randomly selected animals were sacrificed by intravenous injection of a lethal dose of pentobarbital (Euthasol, AST Farma, Oudewater, The Netherlands) at 17 different time points after start of label administration. During necropsy, the left and right pre-scapular LNs and the middle part of the sternum were isolated. Venous blood was collected from the jugular vein in heparinized Vacutainer (BD Biosciences) tubes prior to injection with pentobarbital. Single cell suspensions from LN were obtained by mechanical disruption, and from BM by flushing the sternum. BM cell suspensions were lysed with lysis buffer (155 mM ammonium chloride, 10 mM potassium bicarbonate, 0.1 mM Na2-EDTA, pH = 7.0). Peripheral blood mononuclear cells (PBMCs) were isolated from blood using SepMate-50 tubes (Stemcell Technologies) and Ficoll-Paque Premium (GE Healthcare) following the manufacturer's protocol. The SepMAte-50 tubes were centrifuged at 1,400 g for 20 min. PBMCs were collected, spun down, and washed prior to cell staining and sorting.

### Flow cytometry and cell sorting

BM and LN cell suspensions and PBMCs were stained for extracellular markers using CD4-AF647 (clone 44.38, AbD Serotec), CD8-PE (clone 38.65, AbD Serotec), CD62L (clone DUI-29, WSU) conjugated with pacific blue (PB) (Zenon PB mouse-IgG1 labeling kit, Life Technologies), CCR7-PeCy7 (clone 3D12, BD Biosciences), and CD14-Viogreen (clone TÜK4, Miltenyi Biotec) monoclonal antibodies. For intracellular markers, cells were subsequently fixed, permeabilized (Cytofix/Cytoperm; BD Biosciences), and stained intracellularly with Ki-67-FITC monoclonal antibody (clone B56, BD Biosciences). Washing steps for intracellular staining were performed using Perm/Wash buffer (BD Biosciences). Double positive (CD4^+^CD8^+^) thymocytes were used to determine the positive gate for Ki-67, since double positive thymocytes have a clear population of cycling cells (Supplementary Figure 5A). Cells were analyzed on an LSR-Fortessa flow cytometer using FACS Diva software (BD Biosciences). Cells were sorted with a purity >93% on a FACSAria III cell sorter (BD Biosciences) using FACS Diva software (BD Biosciences). CD62L^+^CCR7^+^ (double positive naive, DP-N) and CD62L^−^CCR7^−^ (double negative memory, DN-M) CD4^+^ and CD8^+^ T-cells were sorted for transcriptome analysis (Supplementary Figure 1A). For deuterium enrichment analysis, cells were stained for CCR7 and CD62L and were sorted based only on CCR7 expression; CCR7^+^ (naive) and CCR7^−^ (memory) CD4^+^ and CD8^+^ lymphocytes were sorted from blood, BM and LN (Supplementary Figures 1B,C). Granulocytes were sorted from lysed whole blood based on their FSC and SSC characteristics and used for deuterium enrichment analysis (Supplementary Figure 1D). CD62L^+^CCR7^+^ (DP-N), CD62L^−^CCR7^−^ (DN-M), CD62L^+^CCR7^−^, CD62L^−^CCR7^+^, total CCR7^−^ (memory) and total CCR7^+^ (naive) CD4^+^ and CD8^+^ T-cells were sorted for functional assays.

### RNA isolation

For RNA isolation, FACS sorted CD62L^−^CCR7^−^ (DN-M), CD62L^+^CCR7^+^ (DP-N) CD4^+^, and CD8^+^ T-cells from blood and CD62L^−^CCR7^−^ (DN-M) CD8^+^ T-cells from BM were sorted, spun down, and stored at −80°C prior to RNA extraction. Before thawing, cells were immersed in QIAzol Lysis Reagent (Qiagen). RNA was isolated and purified using the RNeasy kit (Qiagen). The concentration was measured on a NanoDrop ND-2000 (Thermo Scientific) and RNA integrity was examined using the 2200 TapeStation System with Agilent RNA ScreenTapes (Agilent Technologies).

### Microarray

Total RNA (50 ng) combined with Spike A was used for amplification and labeling according to the Two-Color Microarray-Based Gene Expression Analysis guide using the Low Input Quick Amp Labeling Kit (Agilent Technologies). For the common reference an equimolar pool of all samples was made and amplified similarly as the test samples with the exception that Spike B was used. Synthesized aRNA was purified with the E.Z.N.A. MicroElute RNA Clean Up Kit (OMEGA bio-tek). The yields of aRNA and CyDye incorporation were measured on the NanoDrop ND-2000. An Agilent microarray (8 × 60 k) was custom-designed using the Agilent e-array microarray design tool v.7.6. The array contains 2,726 negative control probes, 1,319 Agilent control probes and 47,151 probes designed on transcripts from the goat (*Capra hircus*) genome GenBank assembly GCF_001704415.1_ARS1_rna transcripts (July 5, 2017) NCBI repository. Hybridization, washing, and scanning were performed according to manufacturer's instructions with an Agilent G2565CA scanner (Agilent Technologies).

### Microarray data processing and normalization

Raw data was read and normalized in R (Version 3.4.0) using the Limma package of Bioconductor. The “Normexp” method with offset = 16, was used for background correction and the resulting data was Quantile Normalized. Empirical Bayes statistics with Benjamini-Hochberg (BH) false discovery rate (FDR) correction was used to obtain statistical output for all to all comparisons.

### IFN-ɤ ELISA

Sorted CD62L^+^CCR7^+^ (DP-N), CD62L^−^CCR7^−^ (DN-M), CD62L^+^CCR7^−^, CD62L^−^CCR7^+^, CCR7^−^ (memory), and CCR7^+^ (naive) CD4^+^ and CD8^+^ T-cells from blood and LN were cultured and stimulated with PMA (20 ng/ml) and ionomycin (1 ng/ml) for 70 h. Supernatant was collected at 20 and 70 h after stimulation, IFN-y production was measured using the BOVIGAM TB IFN-γ ELISA kit (Bovigam). Samples were tested in triplicates. IFN-γ optical density (OD) from unstimulated samples (background) was subtracted from the OD of stimulated samples.

### DNA isolation

Genomic DNA was isolated from CD4^+^CCR7^+^ (naive), CD4^+^CCR7^−^ (memory), CD8^+^CCR7^+^ (naive), and CD8^+^CCR7^−^ (memory) T-cells sorted from blood, LN and BM, and granulocytes using the ReliaPrep Blood gDNA Miniprep System (Promega, Madison, WI, USA) and stored at −20°C before processing for gas chromatography/mass spectrometry (GC/MS).

### Measurement of ^2^H_2_O enrichment in body water and DNA

Deuterium enrichment in plasma and DNA was measured by GC/MS using an Agilent 5973/6890 GC/MS system (Agilent Technologies). Plasma was derivatized to acetylene (C_2_H_2_, M = 26) as previously described (29). The derivative was injected into the GC/MS equipped with a PoraPLOT Q 25 × 0.32 column (Varian), and measured in SIM mode monitoring ions m/z 26 (M+0) and m/z 27 (M+1). From the ratio of ions, plasma deuterium enrichment was calculated by calibration against ^2^H_2_0 standards of known enrichment. DNA obtained from sorted lymphocytes and granulocytes was hydrolyzed to deoxy-ribonucleotides and derivatized to penta-fluoro-triacetate (PFTA, M = 435) (29). The derivative was injected into the GC/MS equipped with a DB-17 column (Agilent Technologies) and measured in SIM mode monitoring ions m/z 435 (M+0), and m/z 436 (M+1). From the ratio of ions, we calculated the deuterium enrichment in the DNA by calibration against deoxyadenosine standards of known enrichment as previously described (6).

### Mathematical modeling of plasma and DNA enrichment data

To control for changing levels of ^2^H in body water over the course of the experiment, a simple label enrichment/decay curve was fitted to ^2^H enrichment in plasma:

(1a) $$\underline{\text{during label intake }(\text{t}\leq \tau ):}S(t)=f(1-e^{-\delta t})$$

(1b) $$\underline{\text{after label intake }(\text{t}>\tau ):}S\left(t\right)=[f\left(1-e^{-\delta t}\right)]e^{-\delta (t-\tau )}$$

as described previously (6) (with minor modification because we did not give an initial boost of label), where *S(t)* represents the fraction of ^2^H_2_O in plasma at time *t* (in days), *f* is the fraction of ^2^H_2_O in the drinking water, labeling was stopped at *t* = τ days, and δ represents the turnover rate of body water per day. The best fit for *S(t)* was used in the labeling equations for the different cell populations (see below). Up- and down-labeling of the granulocyte population was analyzed as previously described (6), to estimate the maximum level of label intake that cells could possibly attain (Supplementary Figure 4 and Supplementary Tables 1, 2). The label enrichment data of all cell subsets were subsequently scaled by the granulocyte asymptote (6).

A mathematical model that allowed for kinetic heterogeneity between cells of the same population was fitted to the labeling data of the different leukocyte subsets. Each kinetic sub-population *i* was modeled to contain a fraction α_i_ of cells with turnover rate *p*_i_. Because we observed that the population sizes hardly changed during the labeling and de-labeling phases of our study (data not shown), we considered a steady state for each kinetic sub-population (i.e., production equals loss), and label enrichment of adenosine in the DNA of each sub-population *i* was modeled by the following differential equation:

(2a) $$\frac{dl_{i}}{dt}=p_{i}cS\left(t\right)\alpha_{i}A-p_{i}l_{i}$$

where *l*_i_ is the total amount of labeled adenosine deoxyribose (dR) in the DNA of sub-population *i* and *A* is the total amount of adenosine in the cell population under investigation, *c* is an amplification factor that needs to be introduced because the adenosine dR moiety contains multiple hydrogen atoms that can be replaced by deuterium (6), and *p*_i_ is the average turnover rate of sub-population *i*. Basically, labeled adenosines in sub-population *i* are gained when a deuterium atom is incorporated with probability *cS*(*t*) in the DNA of cells that replicate at rate *p*_i_, and labeled adenosine is lost when cells of sub-population *i* are lost at rate *p*_i._ For naive T-cells this replication may occur both in the periphery and in the thymus. Scaling this equation by the total amount of adenosine in the DNA of sub-population *i*, i.e., defining *L*_i_ = *l*_i_*/(*α_i_*A)*, yields

(2b) $$\frac{dL_{i}}{dt}=p_{i}cS\left(t\right)-p_{i}L_{i}$$

throughout the up- and down-labeling period, where *L*_i_ represents the fraction of labeled adenosine dR moieties in the DNA of sub-population *i*. The corresponding analytical solutions are

(3a) $$L_{i}(t)=\frac{c}{\delta -p_{i}}[\delta f\left(1-e^{-p_{i}t}\right)-p_{i}f\left(1-e^{-\delta t}\right)]$$

during label intake (*t* ≤ τ), and

(3b) $$L_{i}(t)\;=\;\frac{c}{\delta -p_{i}}[\delta f\left(e^{-p_{i}(t-\tau )}-e^{-p_{i}t}\right)-p_{i}f\left(e^{-\delta (t-\tau )}-e^{-\delta t}\right)]$$

after label intake (*t* > τ).

The fraction of labeled DNA in the total T-cell population under investigation was subsequently derived from *L*(*t*) = ∑α_*i*_*L*_*i*_(*t*), and the average turnover rate *p* was calculated from *p* = ∑α_*i*_*p*_*i*_. Average lifespans were calculated as 1/*p*.

Because all enrichment data were expressed as fractions, labeling data were arcsin(sqrt) transformed before the mathematical model was fitted to the data. We followed a stepwise selection procedure to determine the number of kinetically different subpopulations to include in the model, adding a new kinetically different subpopulation into the model until the average turnover rate no longer significantly changed (4). For populations that appeared to behave kinetically homogeneously, the fitting procedure set the contribution of the extra subpopulation(s) to zero. The labeling curves of CD4^+^ CCR7^−^ (memory) T-cells in blood, LN, and BM as well as CD8^+^ CCR7^−^ (memory) T-cells in blood were significantly better described by a model including two kinetically different subpopulations while the other populations required only one.

### Statistical analysis

Differences between groups were assessed using Wilcoxon signed-rank test (GraphPad, software, Inc., La Jolla, CA, USA). Deuterium-enrichment data were fitted with the function nlm in R. The 95% confidence intervals were determined using a bootstrap method where the residuals to the optimal fit were resampled 500 times. Differences with a *p*-value < 0.05 were considered significant.

## Results

### Flow cytometric characterization of goat blood, BM, and LN-derived T-cells

**Figure 1:**
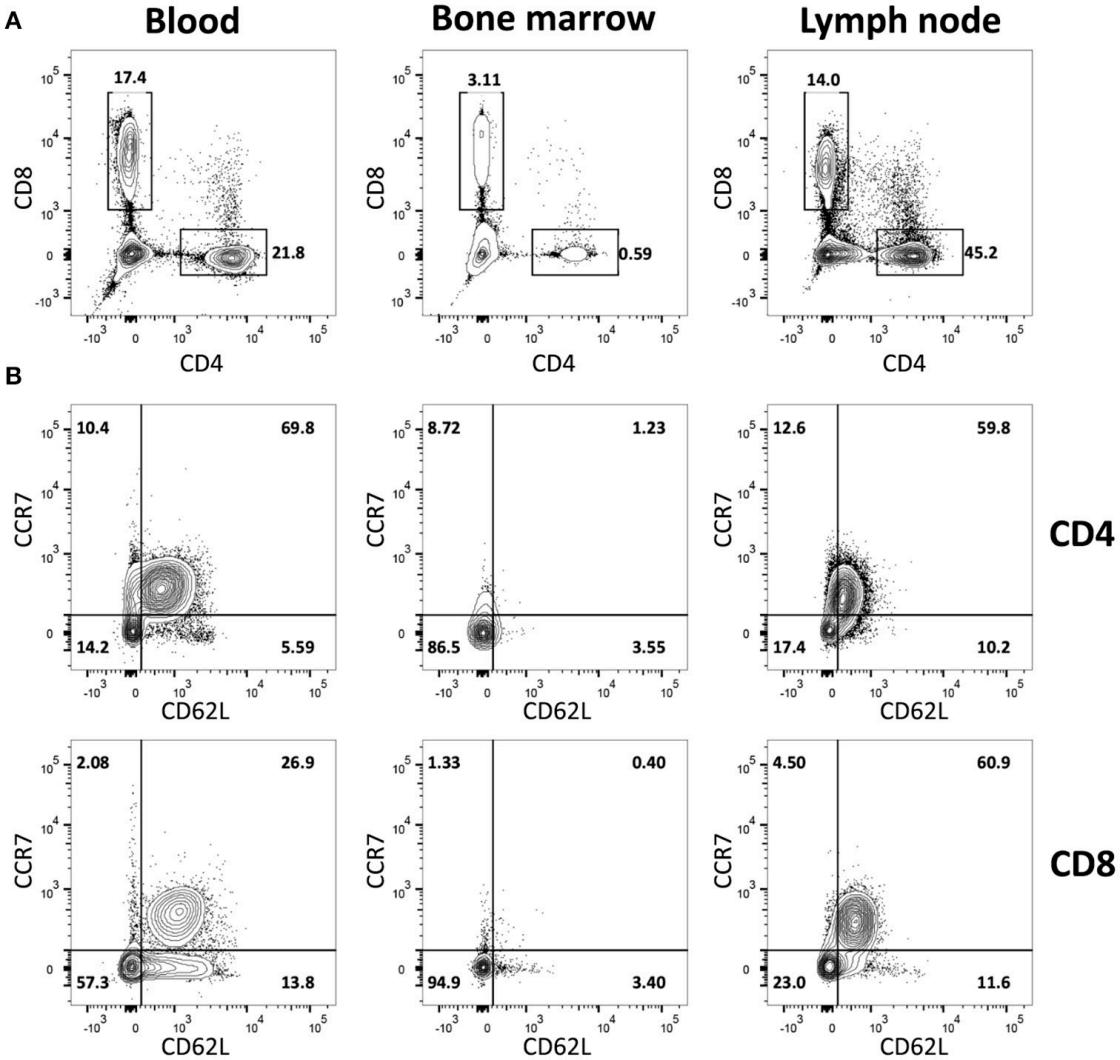
Immunophenotypic characterization of BM-derived T-cells. Blood-, BM- and LN-derived mononuclear cells were obtained from healthy goats. **(A)** Staining of CD4^+^ and CD8^+^ lymphocytes and **(B)** of CD62L^+^ and CCR7^+^ within CD4^+^ *(top row)* and CD8^+^ *(bottom row)* T-cells isolated from blood, BM, and LN of a representative goat.

To study the *in vivo* dynamics of memory T-cells simultaneously in blood, BM and LN, we made use of adult female goats as animal model. Immunophenotypic analysis showed that, in goats, BM lymphocytes include on average 1.4% CD4^+^ and 6.7% CD8^+^ T-cells, much lower percentages than in blood (18.2% CD4^+^ and 24.4% CD8^+^) and LN (31.9% CD4^+^ and 16.4% CD8^+^). BM also presented a lower CD4/CD8 ratio compared to blood and LN (Figure 1A, Table 1). In addition, CD4^+^ and CD8^+^ T-cells from BM consistently expressed lower levels of selectin-L (CD62L) and CC chemokine receptor 7 (CCR7), molecules that facilitate T cell homing to lymphoid tissues, than T-cells from blood and LN, with the majority of BM T-cells being CCR7^−^CD62L^−^ (Figure 1B, Table 1). This flow cytometric characterization suggests that T-cells obtained by flushing the sternum are a phenotypically distinct population from T-cells found in peripheral blood. CD62L and CCR7 are predominantly expressed by naive T-cells cells and their lack of expression is a hallmark of memory T-cells in both human (30) and mouse (31, 32). We focused on these markers, because of the limited availability of monoclonal antibodies for studies in goats, to separately analyse the CCR7^+^CD62L^+^ and the CCR7^−^CD62L^−^ T-cell populations assuming these to be enriched in naive and memory T-cells, respectively.

**Table 1:** Immunophenotypic characterization of BM-derived T-cells.

	**Blood (*****N*** = **18)**		**BM (*****N*** = **20)**		**LN (*****N*** = **18)**	
Lymphocytes	59.0 (17.6)		23.6 (6.7)		69.9 (12.7)	
CD4^+^	18.2 (7.0)		1.4 (1.9)		31.9 (12.2)	
CD8^+^	24.4 (8.1)		6.7 (6.3)		16.4 (4.6)	
CD4/CD8 ratio	0.9 (0.6)		0.2 (0.1)		2.1 (1.0)	
	**CD4**^+^	**CD8**^+^	**CD4**^+^	**CD8**^+^	**CD4**^+^	**CD8**^+^
CCR7^−^ (memory)	40.0 (17.5)	80.2 (13.3)	88.3 (5.7)	97.8 (1.3)	30.2 (7.7)	46.0 (16.1)
CD62L^−^	45.5 (17.1)	64.6 (14.8)	92.7 (4.3)	94.0 (2.6)	41.6 (14.8)	43.0 (13.7)
CCR7^−^CD62L^−^ (DN-M)	26.5 (9.0)	59.8 (13.1)	83.4 (6.7)	92.3 (2.6)	20.7 (6.9)	32.6 (14.0)

*Flow cytometric analysis was used to assess the percentage of blood, LN and BM-derived mononuclear cells expressing CD4, CD8, CCR7, and CD62L. Percentage lymphocytes based on FSC/SSC within the total events, percentage of CD4^+^ and CD8^+^ cells within total lymphocytes, CD4/CD8 ratios, and percentage of CCR7^−^ (memory), CD62L^−^ and CCR7^−^CD62L^−^ (DN-M) cells within the total CD4^+^ and CD8^+^ lymphocyte populations are shown. N = number of animals analyzed. We report mean and standard deviation (SD)*.

### CCR7^−^CD62L^−^ T-cells present transcriptional and functional features of memory T-cells

**Figure 2:**
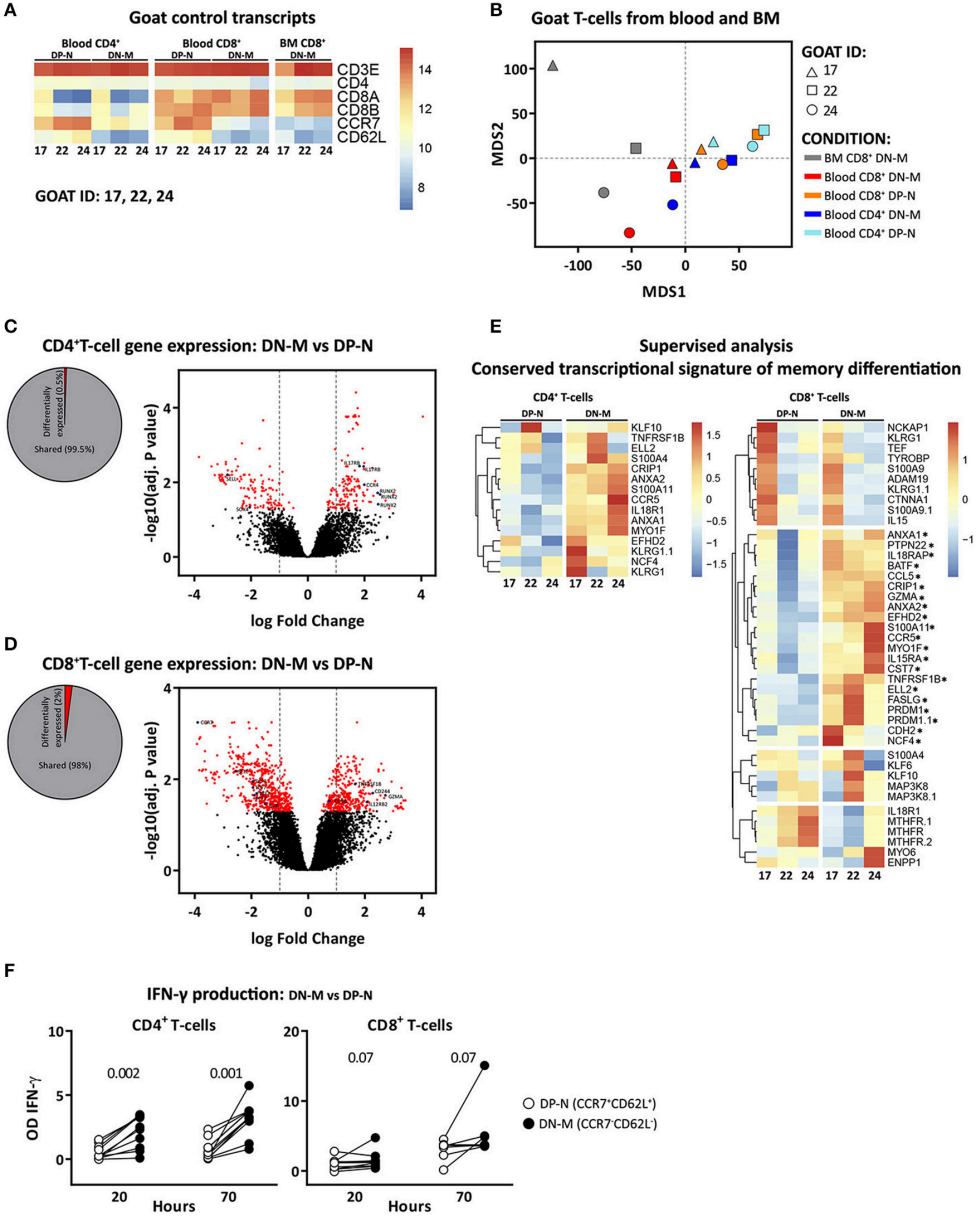
CCR7^−^CD62L^−^ (DN-M) T-cells present transcriptional and functional characteristics of memory T-cells. Microarray profiling was performed on DN-M (CCR7^−^CD62L^−^) and DP-N (CCR7^+^CD62L^+^) CD4^+^ and CD8^+^ T-cells from blood and DN-M CD8^+^ T-cells from BM of 3 goats (goat 17, 22, and 24). **(A)** Heatmap showing normalized expression levels of control genes, CD3E, CD4, CD8A, CD8B, CCR7, and CD62L for all the samples. **(B)** Multidimensional scaling (MDS) of DN-M and DP-N samples from blood and BM for CD4^+^ and CD8^+^ T-cell subsets, based on the global transcriptome (~47,151 probes). **(C**,**D)** Diagram showing the percentage significantly differentially expressed genes (adjusted *p*-value (BH) < 0.05) between DN-M and DP-N CD4^+^ **(C)** or CD8^+^ **(D)** T-cells from blood, as well as volcano plots illustrating the log_2_ fold change differences in gene expression levels between DN-M and DP-N CD4^+^ **(C)** or CD8^+^ **(D)** T-cells from blood. Significantly differentially expressed genes (adjusted p-value (BH) < 0.05) are shown in red, blue dots depict genes related to memory differentiation. **(E)** Heatmap showing the normalized expression of genes from the adaptive memory signature (33) in CD4^+^ T-cells *(left panel)*; and of genes from the conserved CD8 memory signature (33) in CD8^+^ T-cells *(right panel)*. Genes up-regulated in DN-M compared to DP-N CD8^+^ T-cells in the 3 different goats are marked with an*. Gene expression is scaled per row. **(F)** DN-M (CCR7^−^CD62L^−^) and DP-N (CCR7^+^CD62L^+^) CD4^+^ and CD8^+^ T-cells sorted from blood were cultured *in vitro* for 70 h in the presence of PMA/ionomycin. Mean IFN-γ production, measured from the supernatant by ELISA, at 20 and 70 h after stimulation is shown as the OD of stimulated samples minus the OD of the background (unstimulated sample). *P*-values obtained using the Wilcoxon signed-rank test are shown.

To validate the memory and naive phenotype of goat T-cells, we performed microarray based gene expression analysis and IFN-γ release analysis on CCR7^−^CD62L^−^ (double negative memory, in short DN-M), cell subset likely enriched for memory T-cells, and CCR7^+^CD62L^+^ (double positive naive, in short DP-N), cell subset likely enriched for naive T-cells, T-cells. *Transcriptome* analysis on DN-M and DP-N CD4^+^ T-cells from blood and CD8^+^ T-cells from blood and BM confirmed at transcriptional level the expression of CD4, CD8A, CD8B, CCR7, and CD62L, and showed high expression of CD3E in all the samples despite the fact that CD4^+^ and CD8^+^ T-cells were not sorted based on CD3, because anti CD3 antibody is not available for goat (Figure 2A). Multidimensional scaling (MDS) was used to visualize all the expression data. In an MDS geometrical plot, distance between points reflects similarity between samples. In the MDS plot, samples segregated by cell type and organ of origin. Within CD4^+^ T-cells, the DP-N and DN-M populations clustered separately; within the CD8^+^ T-cell subset, DN-M cells also clustered together and separately from DP-N cells. CD8^+^ DN-M T-cells from BM origin clustered together and showed more similarity to blood CD8^+^ DN-M than to CD8^+^ DP-N T-cells (Figure 2B). Taken together, these results suggest that CCR7 and CD62L expression define transcriptionally distinct T-cell subsets.

Differential gene expression analysis showed, in agreement with gene expression profiles from human and mouse (34), that a small percentage of genes were expressed at significantly different levels between memory and naive T-cells. Applying the criteria for significance, for CD4^+^ samples, we identified 262 differentially expressed genes [adjusted *p*-value (BH) < 0.05], corresponding to 0.5% of the total (Figure 2C). The differentially expressed genes with an absolute log_2_ fold change ≥ 1 included genes previously identified to play a role in the differentiation from naive to memory, such as *IL17RB, CCR4* (35) and *RUNX2* (36), which were up-regulated, and *SOX4* and *Bach2* (36) which were down-regulated in DN-M as compared to DP-N CD4^+^ T-cells (Figure 2C, Supplementary Table 3). Memory and naive CD8^+^ T-cells generally show more differentially expressed genes than CD4^+^ T-cells (34); in accordance, we identified 984 differentially expressed genes (2% of the total) between DN-M and DP-N CD8^+^ T-cells (Figure 2D). Significantly up-regulated genes (log_2_ fold change ≥1) in DN-M compared to DP-N CD8^+^ T-cells included genes promoting T-cell survival and homeostasis, including *TNFS1B* and *IL12RB2*, molecules involved in immune activation, such as CD58, and genes involved in the cytotoxic effector function of T-cells, like *GZMA* and *CD244* (34). *IL7R* and *TCF7*, genes involved in naive T-cell maintenance (37, 38), were down-regulated in DN-M CD8^+^ T-cells (Figure 2D, Supplementary Table 3). For a supervised analysis, we used the conserved transcriptional signature of CD4^+^ and CD8^+^ memory T-cell differentiation describing the genes that are up-regulated in memory compared to naive T-cells in both human and mice (33). Goat CD4^+^ DN-M T-cells showed up-regulated expression of all the genes composing the adaptive memory signature. For CD8^+^ T-cells, we found that the conserved CD8 memory signature described by Haining et al. (33) was enriched in goat CD8^+^ DN-M T-cells. Twenty out of 36 genes were up-regulated in CD8^+^ DN-M T-cells in the 3 different goats (Figure 2E).

Finally, to *functionally* validate the memory phenotype of CCR7^−^CD62L^−^ T-cells in goats, we analyzed the ability of DN-M and DP-N T-cells to produce IFN-γ after stimulation (see methods). We found that 70 h after stimulation, CD4^+^ and CD8^+^ DN-M T-cells were able to produce higher amounts of IFN-γ than their DP-N counterparts. In addition, DN-M T-cells reacted faster to stimulation, as a substantial amount of IFN-γ production was already detected at 20 h (Figure 2F). Altogether, these data support the interpretation that in goats CCR7^−^CD62L^−^ (DN-M) T-cells are memory T-cells. In fact, we found the production of IFN-γ to be reliant on the expression of CCR7, not on CD62L, as CCR7^−^CD62L^+^ T-cells produced similar amounts of IFN-γ as CCR7^−^CD62L^−^ and CCR7^−^ T-cells, and higher amounts than CCR7^+^CD62L^+^ and CCR7^+^ T-cells (Supplementary Figure 2). Given that just a small fraction of the total population of CCR7^−^ T-cells expressed CD62L, we therefore focused the kinetic analyses on the CCR7^−^ population (from now on referred to as memory cells).

### Memory T-cells in BM and LN contain lower percentage of Ki-67 positive cells than memory T-cells in blood

**Figure 3:**
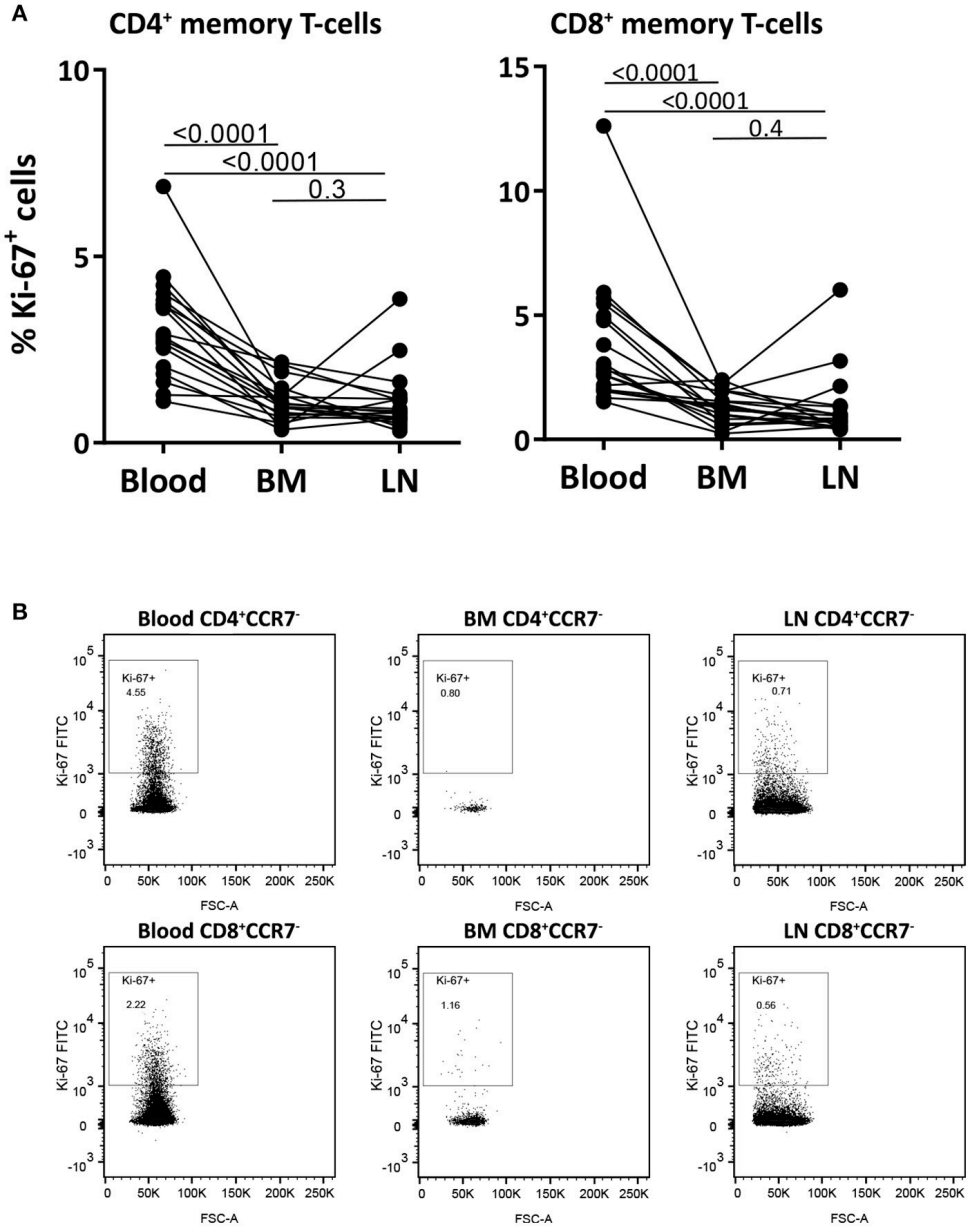
Memory T-cells from blood have higher percentages of Ki-67 positive cells than those from BM and LN. **(A)** The fraction of memory (CCR7^−^) CD4^+^ and CD8^+^ T-cells expressing the proliferation marker Ki-67 was assessed in paired samples from blood, BM, and LN. Paired samples were compared using the Wilcoxon signed-rank test, *p*-values are shown. **(B)** Intracellular Ki-67 staining of CD4^+^ and CD8^+^ memory (CCR7^−^) T-cells isolated from blood, BM, and LN of a representative goat.

To study the dynamics of memory T-cells in the different compartments, we first measured the percentage of Ki-67 positive cells in paired samples from blood, BM and LN CD4^+^ and CD8^+^ memory (CCR7^−^) T-cells. Ki-67 is a nuclear protein expressed during all phases of the cell cycle except for G_0_, thus actively dividing and recently divided cells express high levels of Ki-67. The percentage of Ki-67^+^ cells was significantly lower in memory T-cells from BM and LN compared to those in blood (*p*-values < 0.0001), with an average fraction of Ki-67^+^ cells of 3.2% of CD4^+^ and 3.9% of CD8^+^ memory T-cells from blood, 1.1% of CD4^+^ and 1.3% of CD8^+^ memory T-cells from BM, and 1.1% of CD4^+^ and 1.3% of CD8^+^ memory T-cells from LN (Figures 3A,B and Supplementary Figure 5). These results are in agreement with previous reports suggesting that memory T-cells in BM show less signs of active cell-division than their counterparts in blood (18, 19, 24).

### Memory T-cells from blood, BM and LN have similar turnover rates

Low percentages of Ki-67 positive cells in memory T-cells from BM have been interpreted as a sign that BM is the place where long-lived memory T-cells reside (18). However, Ki-67 inherently provides no information on the longevity of the cells. We therefore used *in vivo* deuterium labeling to quantify the turnover of memory T-cells from blood, BM, and LN. Animals received ^2^H_2_O for 4 weeks and were sacrificed at different time points during the labeling and the subsequent de-labeling period, such that a cross-sectional up- and down-labeling curve of deuterium enrichment could be constructed. Using a mathematical model that takes into account the possible kinetic heterogeneity of a cell population (4), we estimated the average turnover rate (*p*) of the different cell populations, i.e., the fraction of cells replaced by new cells per day, and deduced the corresponding average lifespan (*1/p*) of the cells in that populations (see material and methods).

**Figure 4:**
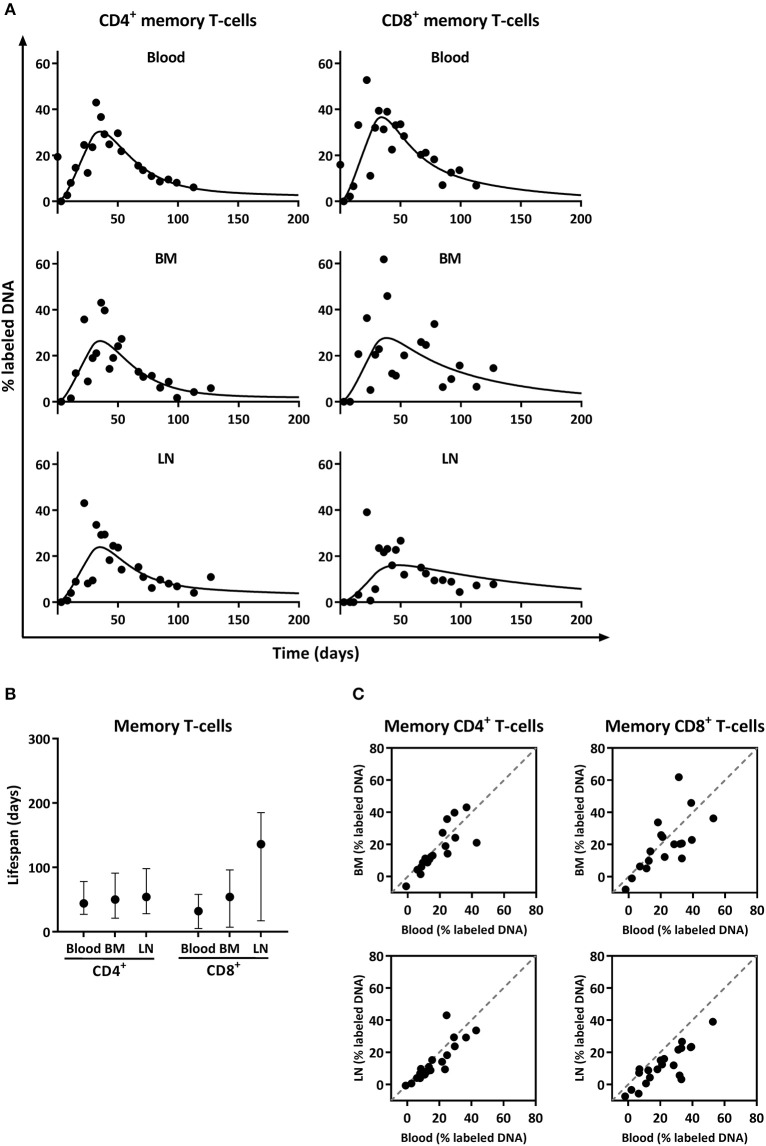
Analysis of deuterium enrichment and summary of the estimated lifespan of CD4^+^ and CD8^+^ memory T-cells from blood, BM and LN. **(A)** Best fits to the level of deuterium enrichment measured in the DNA of CD4^+^ and CD8^+^ memory (CCR7^−^) T-cells from blood, BM and LN. Label enrichment in the DNA was scaled between 0 and 100% by normalizing to the maximum enrichment in granulocytes (See material and methods). **(B)** Estimated lifespans of CD4^+^ and CD8^+^ memory T-cells in days, and their respective 95% confidence limits. **(C)** Correlation between deuterium enrichment in BM and blood, and LN and blood. The gray dashed line represents the X = Y line.

Despite the observed differences in the percentage of Ki-67 positive cells, deuterium enrichment levels in CD4^+^ and CD8^+^ memory (CCR7^−^) T-cells from blood, BM, and LN were very similar (Figure 4A). The fits of the model to the experimental data (Figure 4A) and their corresponding estimates revealed no significant differences in the average turnover rates of memory T-cells isolated from BM and blood (Figure 4B). The estimated average lifespan of memory T-cells isolated from BM was 50 days [(95% confidence interval (CI) = 21;91] for CD4^+^ and 54 days (CI = 7;96) for CD8^+^ cells. Memory T-cells obtained from blood had an estimated average lifespan of 44 days (CI = 27;78) for CD4^+^ and 32 days (CI = 5;58) for CD8^+^ (Figure 4B). For the LN, we estimated that memory CD4^+^ T-cells live on average 54 days (CI = 28;98), and CD8^+^ T-cells 136 days (CI = 17;185) (Figure 4B). The labeling curves of memory CD4^+^ T-cells isolated from the blood, LN, and BM and of memory CD8^+^ T-cells isolated from the blood were significantly better described by a model including two kinetically different subpopulations, while the labeling curves of memory CD8^+^ T-cells from the LN and BM were well described by a kinetically homogeneous model.

Deuterium enrichment in the DNA of memory CD4^+^ and CD8^+^ T-cells highly correlated between BM and blood, as well as between LN and blood (both *R*^2^ > 0.70) and all data points were close to the x = y line (Figure 4C). These results suggest that, despite the observed differences in the fraction of Ki-67 positive cells, the turnover rates of CD4^+^ and CD8^+^ memory T-cells obtained from blood, BM, and LN are very similar, and that the vast majority of memory T-cells, even the ones located in BM and LN, are short lived, with an average lifespan of about 50 days.

Of note, both the percentage of Ki-67 positive cells (*p*-values < 0.0001, Supplementary Figures 3A,B), and the level of deuterium incorporation (Supplementary Figures 3C,D) were higher in memory (CCR7^−^) compared to naive (CCR7^+^) T-cells, in line with the typical observation in mice and humans that memory T-cells express higher levels of Ki-67 and reach higher deuterium enrichment than naive T-cells (4, 39).

### Memory T-cells from BM do not share the tissue resident memory (TRM) transcriptional signature

**Figure 5:**
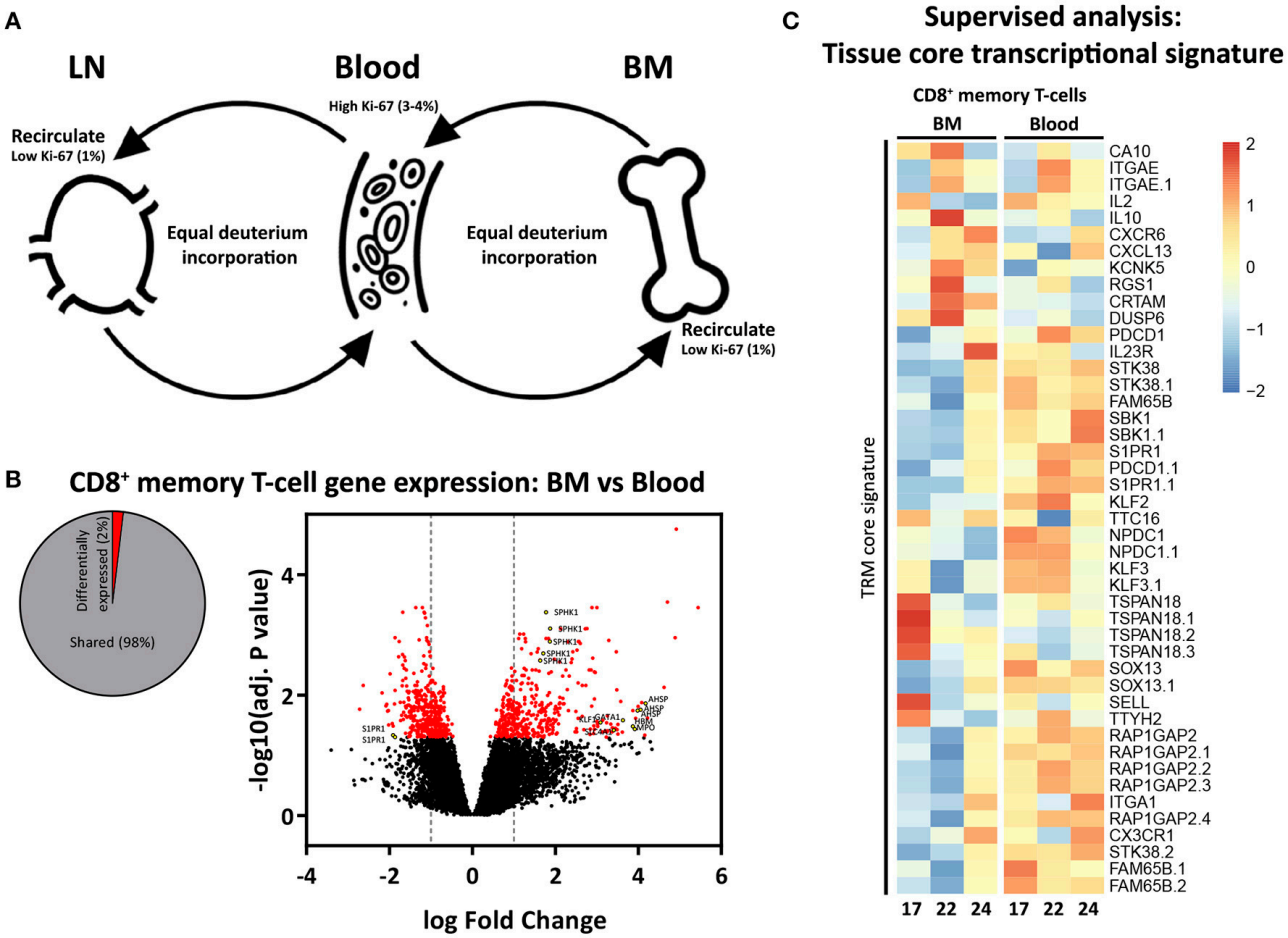
Proposed model of proliferation and recirculation. **(A)** Memory T-cells from BM and LN have a lower fraction of Ki-67 positive cells compared to memory T-cells from blood, but similar deuterium incorporation. We here propose a dynamic model in which memory T-cells are continuously recirculating between BM, blood, and LN. If BM memory T-cells would compose a separate population of resting and resident cells, one would expect to find low percentages of Ki-67 as well as low deuterium incorporation in BM. The fact that memory T-cells isolated from the BM had substantial levels of deuterium enrichment in the DNA shows that memory T-cells from BM do proliferate. The fact that the percentage of Ki-67 positive cells in BM was lower compared to blood while deuterium enrichment curves were very similar strongly suggests that memory T-cells are recirculating. Because of the difficulties in the interpretation of Ki-67 expression (see discussion) it remains unclear where the divisions occur. **(B)** Diagram showing the percentage significantly differentially expressed genes (adjusted *p*-value (BH) < 0.05) between BM and blood memory (CCR7^−^) CD8^+^ T-cells, as well as a volcano plot illustrating the log_2_ fold change differences in gene expression. Significantly differentially expressed genes are shown in red, yellow dots depict tissue related genes. **(C)** Heatmap showing normalized expression levels of genes defining the tissue resident memory (TRM) core transcriptional signature (42). Gene expression is scaled per row.

The most parsimonious explanation for the opposing Ki-67 and deuterium results would be that memory T-cells are constantly cycling and circulating between BM, blood, and LN (40, 41), and that memory T-cells may pick up deuterium while dividing outside the BM (Figure 5A). We hypothesized that, if memory T-cells in BM would belong to a population of circulating T-cells, they would not share the TRM core transcriptional signature defined for human and mouse lymphocytes (42). While we found 2% differentially expressed genes between CD8^+^ memory T-cells from BM and blood (Figure 5B), we did not find any enrichment for genes defining the TRM core transcriptional signature (Figure 5C), supporting our hypothesis that the vast majority of BM memory T-cells are not sessile and continuously recirculate.

## Discussion

Our 4-week *in vivo* ^2^H_2_O labeling study suggests that, in goats, memory T-cells from BM are maintained by continuous low-level proliferation. Both for CD4^+^ and CD8^+^ T-cells, we found no significant differences in deuterium labeling, and hence in cellular lifespans, between memory T-cells isolated from the blood, BM, and LN, while the percentage of Ki-67 positive cells differed significantly. The finding that the fraction of Ki-67 positive cells was smaller for memory T-cells isolated from the BM compared to memory T-cells from the blood is in line with previous findings in mice and humans (18, 19, 24). Our *in vivo* deuterium labeling data demonstrate, however, that these differences in Ki-67 expression should not be interpreted as a sign that BM memory T-cells are long-lived. Our data support the view that memory T-cells in blood, BM, and LN are part of a dynamic system, in which the vast majority of cells are maintained by self-renewal and continuously recirculate (Figure 5A).

The goat as an animal model enabled us to simultaneously compare memory T-cell dynamics in blood, BM, and LN. One major disadvantage of this model is that T-cell subsets in goats are less well characterized than in mice and humans. We here showed that, both for CD4^+^ and CD8^+^ T-cells, CCR7^−^ T-cells present phenotypic (Figure 1B and Table 1), functional (Figure 2F), transcriptional (Figures 2C–E) and kinetic (Figures 3, 4 and Supplementary Figure 3) characteristics of memory T-cells and are distinct from CCR7^+^ T-cells, which present naive-like features. In line with observations in mice and humans (18–20, 41, 43–46), the BM of goats is composed of lower percentages of CD4^+^ and CD8^+^ T-cells than blood and LN, and is enriched in memory T-cells. The differentially expressed genes observed between BM and blood support that CD8^+^ memory T-cells isolated from BM are a separate population and are not just cells sampled from the small blood vessels in BM (Figure 5B). An advantage of this model system is that these outbred animals were routinely vaccinated at young age and exposed to a broad range of pathogens throughout life. It has recently been shown that the memory T-cell compartment of mice exposed to pathogens, unlike that of clean laboratory mice, contains a lot more memory T-cells and thereby resembles that of adult humans (47), and that the BM T-cell composition dramatically changes toward a memory phenotype upon infection and pathogen clearance (19). The conventional environment to which goats were exposed prior to and during the study likely led to a more mature and adult human-like immune system.

Our conclusion that BM memory T-cells must be recirculating through the body is supported by *in situ* BM labeling studies (40) and parabiosis experiments (41), which have shown more directly that memory T-cells migrate in and out of the BM. The fact that short-pulse BrdU labeling resulted in relatively high BrdU incorporation in memory T-cells from BM, while longer BrdU administration led to similar BrdU incorporation in memory T-cells from different organs, also supports the view that memory T-cells recirculate between BM and the other compartments (17). In addition, it has been shown that BM contains highly permeable vessels that are restricted to immature and mature leukocyte migration, suggesting that BM facilitates leukocyte trafficking (48). This is in contrast to studies that have shown that a significant fraction, but not the vast majority, of memory T-cells in BM express CD69, a molecule implicated in tissue retention. Such studies have proposed that memory T-cells reside in BM and do not migrate to other organs (18, 24).

Whether memory T-cells in BM are maintained by continuous cell division or by cellular longevity is also heavily debated (16, 49). Notably, in mouse studies, memory T-cell kinetics in BM are typically compared to those of the spleen and LN, while human studies generally base their comparisons on memory T-cells from blood due to the difficulty in accessing peripheral organs, such as LN and spleen. Our finding that the percentage of Ki-67 positive cells is consistently higher in blood than in BM and LNs is in line with previous studies (18, 50), and illustrates that the source of T-cells taken as a reference has an impact on data interpretation. When comparing Ki-67 expression levels between BM and blood, one could conclude that memory T-cells in BM are resting in terms of proliferation; however, the opposite conclusion would be reached when comparing Ki-67 expression between BM and LN. We overcame this problem by simultaneously comparing the kinetics of memory T-cells from BM, blood and LN.

The dispute in the literature may also be partially due to the different techniques used to measure cell dynamics. For CD8^+^ memory T-cells, studies based on DNA content analysis (17, 25), BrdU labeling (17, 25, 27, 46, 51, 52) and CFSE labeling (17, 27, 51, 53, 54) have all suggested that in mice the division rate of memory T-cells in BM is greater than in spleen and LN. The fact that all three techniques gave similar results strongly suggests that the BM is the preferential place for memory T-cells to divide. However, separately each technique has its caveats, as outlined in the introduction. Meanwhile, other studies have proposed that memory T-cells from BM are resting in terms of proliferation, as they have shown that CD8^+^ memory T-cells from BM express lower percentages of Ki-67 than their blood (18) and spleen counterparts (19, 24). Based on such low expression levels of Ki-67 in BM, and the fact that *ex vivo*-isolated BM memory T-cells are transcriptionally less active than the *in vitro*-stimulated memory T-cells (24), it has been proposed that many memory T-cells in BM are resting in G_0_ of the cell cycle and are, hence, long lived. This hypothesis has also been supported by a recent study showing that absolute numbers of CD8^+^ memory lymphocytes in BM are unaffected by cyclophosphamide, an alkylating agent that cross-links DNA leading to apoptosis of cells that attempt to divide, while half of the CD8^+^ memory T-cells in spleen die during cyclophosphamide treatment (55). Although it remains very puzzling how to reconcile these findings with our own results, the techniques used in these studies also have some limitations. First, as mentioned before, Ki-67 expression and DNA content analysis are static measurements of cell proliferation. Second, since DNA cross-linking by cyclophosphamide leads to an enormous loss of cells, this could change the dynamics of the remaining cells in response to the lymphopenia that is induced (56–59).

Our current *in vivo* labeling results strongly suggest that memory T-cells from BM, as well as from blood and LN, are relatively short-lived. Mathematical modeling suggested that the memory CD4^+^ T-cell pools in blood, LN, and BM, and the memory CD8^+^ T-cell pool in blood are composed of at least two 2 kinetically different subpopulations, as previously reported for memory T-cell populations in mice and humans (4). These kinetically different subpopulations may reflect phenotypically different subsets (e.g., effector memory and central memory T-cells), and/or subsets that differ in the exposure to their cognate antigen. Indeed, it has recently been shown that yellow-fever-virus antigen-specific memory T-cells have longer lifespans than the bulk of memory phenotype T-cells (60). Although we cannot formally exclude the possibility that a subpopulation of memory cells may be composed of very long-lived cells that failed to pick up deuterium during the 4 week labeling period, our data convincingly show that, if present, such a population does not preferentially reside in the BM. To formally prove this, a long-term *in vivo* labeling experiment would have to be designed.

All studies based on Ki-67 expression (18, 19, 24), including ours, have reported lower Ki-67 expression of memory T-cells in BM when compared to those in blood and LN. Although the relatively high Ki-67 expression in blood seems to suggest that memory T-cells preferentially divide in circulation rather than in BM or LN, we consider this unlikely. The induction of Ki-67 expression corresponds to the entry of resting cells into the cell cycle, however its expression can be maintained up to 7 days after the completion of mitosis (61–63). This suggests that Ki-67 may be a good indicator of recent cell-cycle activity but may not be a marker for active cell division. We think that blood may be enriched for Ki-67 positive T-cells that have recently divided elsewhere, and may not necessarily have undergone their cell division in blood. One possible explanation is that upon division in the LN, T-cells preferentially egress to blood, which is in line with the observation that recently activated and expanded T-cells egress rapidly from the LN (64). This hypothesis is also supported by the observation that a higher proportion of BrdU labeled cells is found in the lymph nodes immediately after labeling, before BrdU levels in blood and LN reach similar levels (65). Although solving this issue is beyond the scope of the article, this again illustrates the limitations in interpreting data based on snapshot markers such as Ki-67.

Taken together, we here found no evidence for a long lifespan of either CD4^+^ or CD8^+^ memory T-cells in BM. Although all reviewed studies convincingly approached the dynamics of BM memory T-cells, the use of different techniques and the comparison to different organs might have led to conflicting results. Because we simultaneously analyzed memory T-cell kinetics in BM, blood and LN using *in vivo* labeling, we conclude that BM memory T-cells do not form a separate population of long-lived cells. In order to translate this fundamental finding to the human situation, a similar *in vivo* deuterium labeling experiment would have to be done in humans. Given that different techniques provide seemingly opposing results, further research is needed not only to address the role of BM in the maintenance of memory T-cells, but also to better understand how to interpret results obtained using different experimental techniques to study lymphocyte turnover and whether clearer insights can be achieved by combining them.

## Data availability statement

The datasets generated for this study can be found in the Gene Expression Omnibus (GEO), accession number GSE119116.

## Author contributions

MB-P, MV, AK, JD, KT, and JB wrote the manuscript. MB-P, LR, AK, KT, and JB designed the experiments. MB-P, MV, LR, and AK performed the experiments. MB-P and MV analyzed the data. JD, RdB, and JB performed mathematical modeling.

### Conflict of interest statement

The authors declare that the research was conducted in the absence of any commercial or financial relationships that could be construed as a potential conflict of interest.
