# Supplementary material for: Short Lifespans of Memory T-cells in Bone Marrow, Blood, and Lymph Nodes Suggest That T-cell Memory Is Maintained by Continuous Self-Renewal of Recirculating Cells
Source: Front Immunol. 2018 Sep 11;9:2054. doi: 10.3389/fimmu.2018.02054 (PMC6141715; doi:10.3389/fimmu.2018.02054)
Supplement: Supplementary file 1 [file Data_Sheet_1.pdf]

Supplementary Material

Short lifespans of memory T-cells in bone marrow, blood and lymph nodes suggest that T-cell memory is maintained by continuous self-renewal of recirculating cells

Mariona Baliu-Piqué, Myrddin Verheij, Julia Drylewicz, Lars Ravesloot, Rob J. de Boer, Ad Koets[#], Kiki Tesselaar[#], José A.M. Borghans[*#]

[#] These authors contributed equally to this work

*** Correspondence:**
J.A.M. Borghans
J.Borghans@umcutrecht.nl

1 Supplementary Figures and Tables

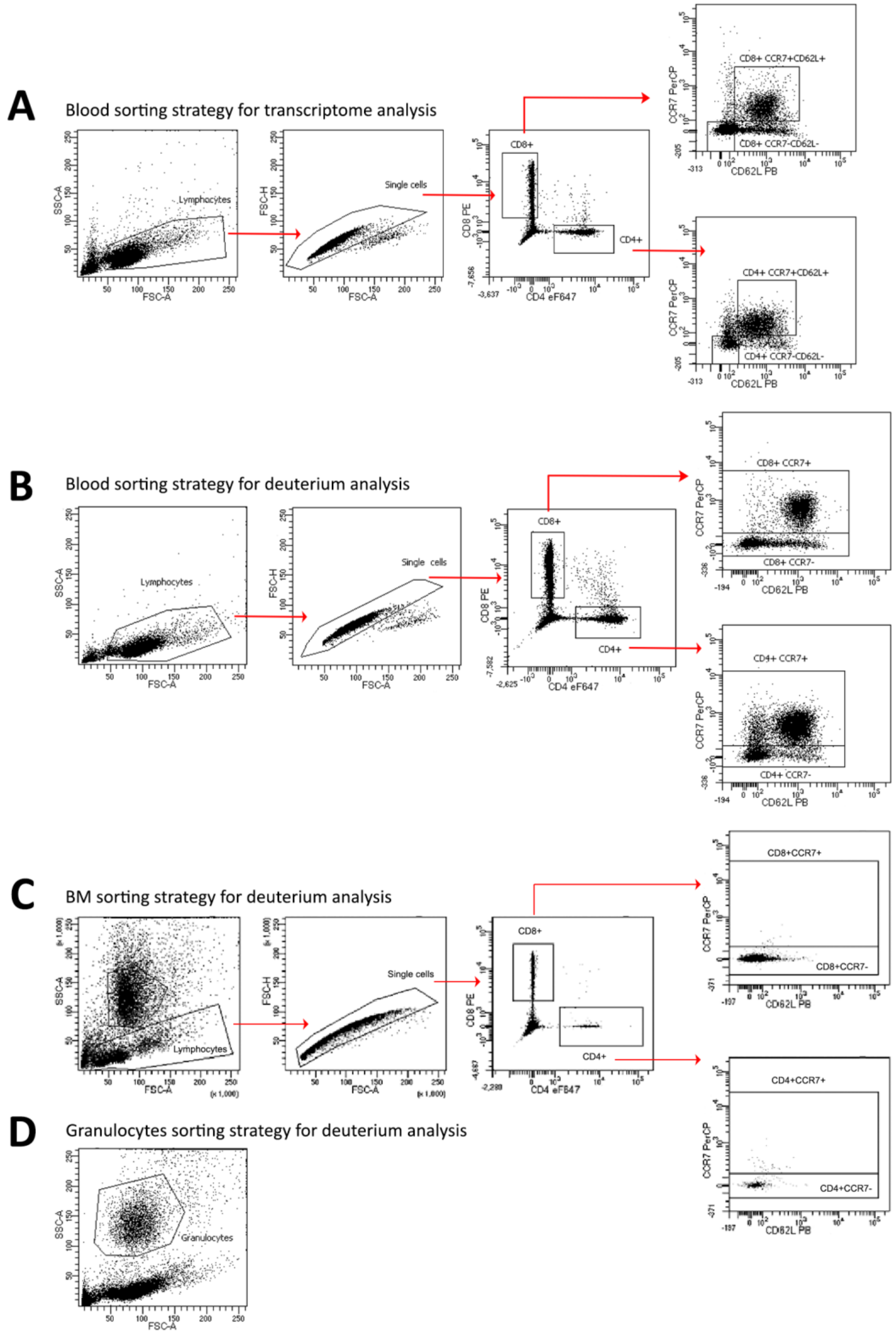

Sup. Figure 1. Sorting strategy for (A) transcriptome analysis, for deuterium enrichment analysis of (B) T-cells from blood, (C) T-cells from BM, and (D) granulocytes from blood.

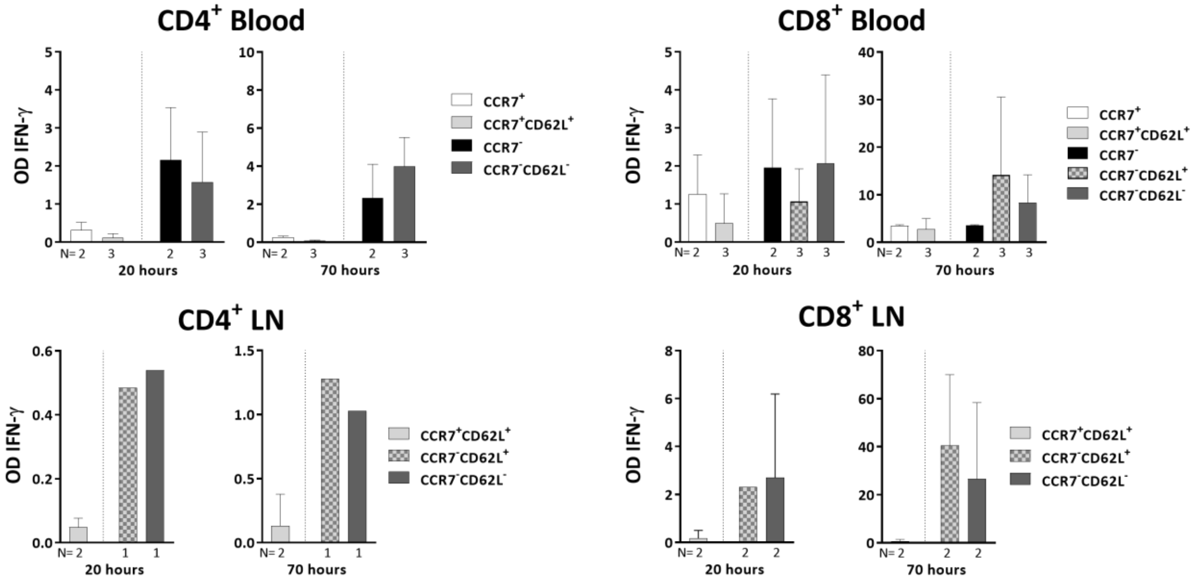

Sup. Figure 2. CCR7$^-$ (memory) T-cells produce higher IFN-γ than CCR7$^+$ (naive) T-cells. Median IFN-γ production for sorted CD4$^+$ and CD8$^+$ CCR7$^+$CD62L$^+$ (DP-N), CCR7$^-$CD62L$^+$, CCR7$^-$CD62L$^-$ (DN-M), CCR7$^+$ (naive) and CCR7$^-$ (memory) T-cells from blood and LN stimulated for 20 and 70 hours with PMA/ionomycin. IFN-γ production is shown as the OD of stimulated samples minus the OD of background. Error bars represent ranges. N=1-3.

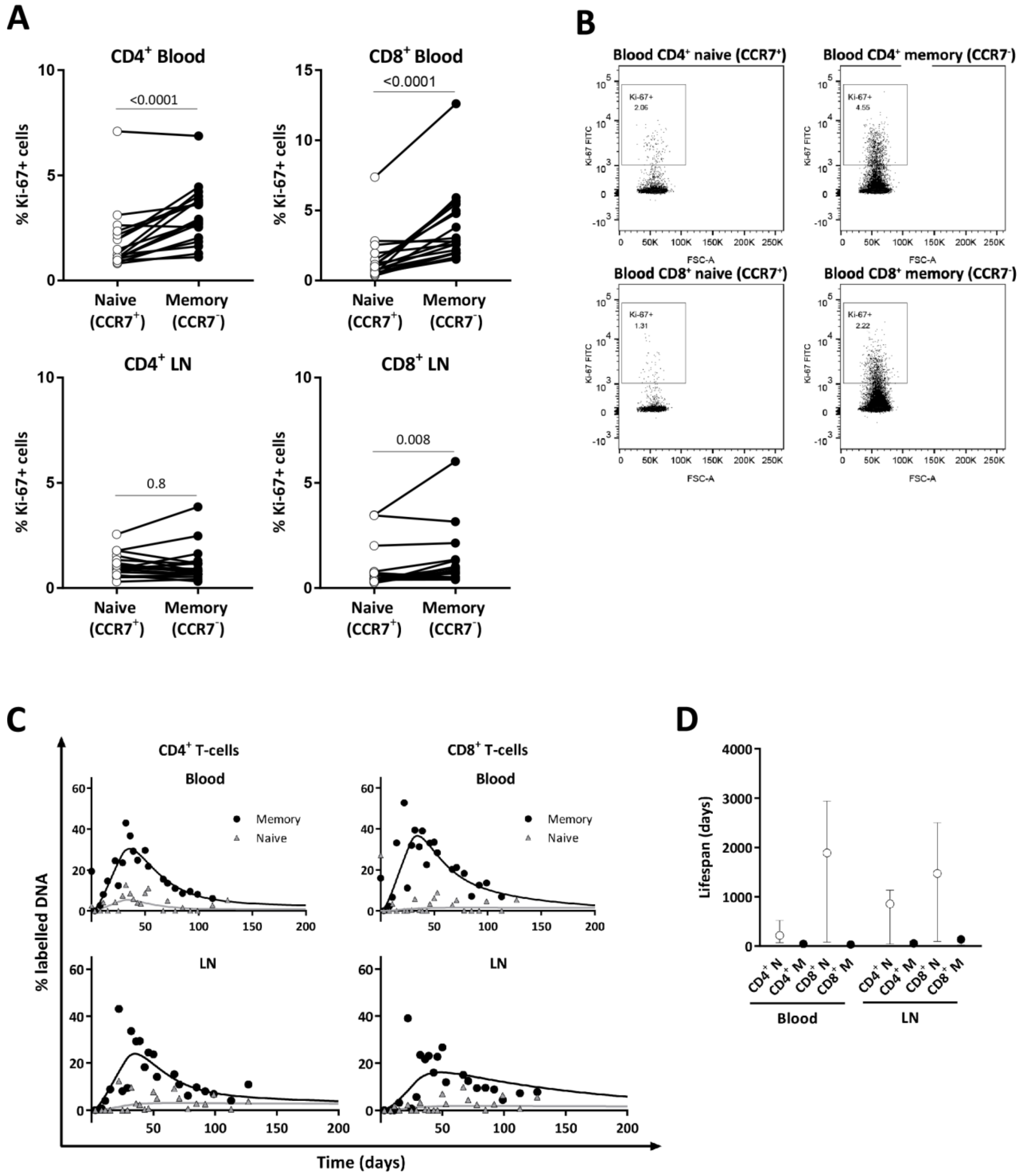

Sup. Figure 3. Memory T-cells (CCR7$^-$) have higher percentage of Ki-67 positive cells and reach higher deuterium enrichment than naive T-cells (CCR7$^+$). (A) Percentage Ki-67 positive cells in memory and naive CD4$^+$ and CD8$^+$ T-cells from blood and LN. Samples were compared using the Wilcoxon signed-rank test, p-values are shown. **(B)** Intracellular Ki-67 staining of CD4$^+$ and CD8$^+$ memory (CCR7$^-$) and naive (CCR7$^+$) T-cells from blood of a

representative goat. **(C)** Best fits to the level of deuterium enrichment measured in the DNA of $CD4^+$ and $CD8^+$ memory ($CCR7^-$) and naive ($CCR7^+$) T-cells from blood and LN. Label enrichment in the DNA was scaled between 0 and 100% by normalizing to the maximum enrichment in granulocytes (See material and methods). Data for memory T-cells (black dots) is also shown in Figure 3 and Figure 4. **(D)** Estimated lifespans in days of $CD4^+$ and $CD8^+$ memory ($CCR7^-$) and naive ($CCR7^+$) T-cells from blood and LN, and their respective 95% confidence limits.

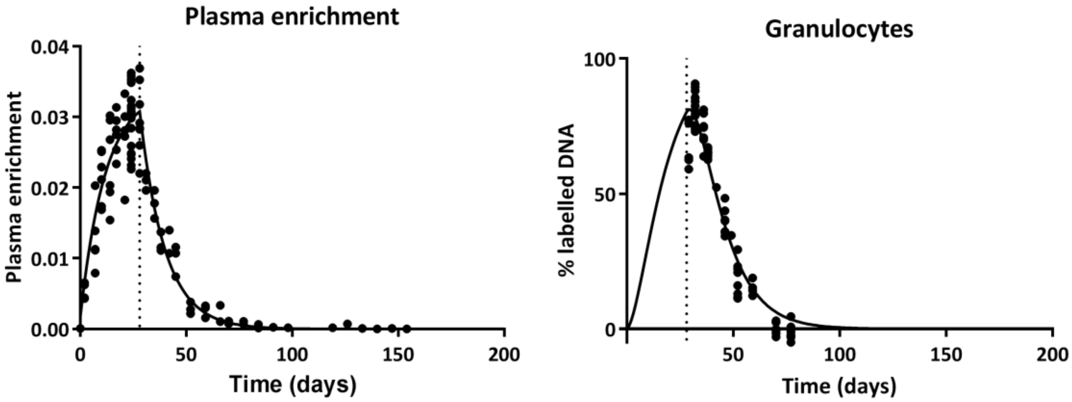

Sup. Figure 4. Best fit of the deuterium enrichment in plasma and granulocytes. Symbols represent individual measurements during the up- and down-labelling phases. Granulocytes were used to normalize the data (see material and methods). Parameter estimates for the best fit are given in Sup. Table 1.

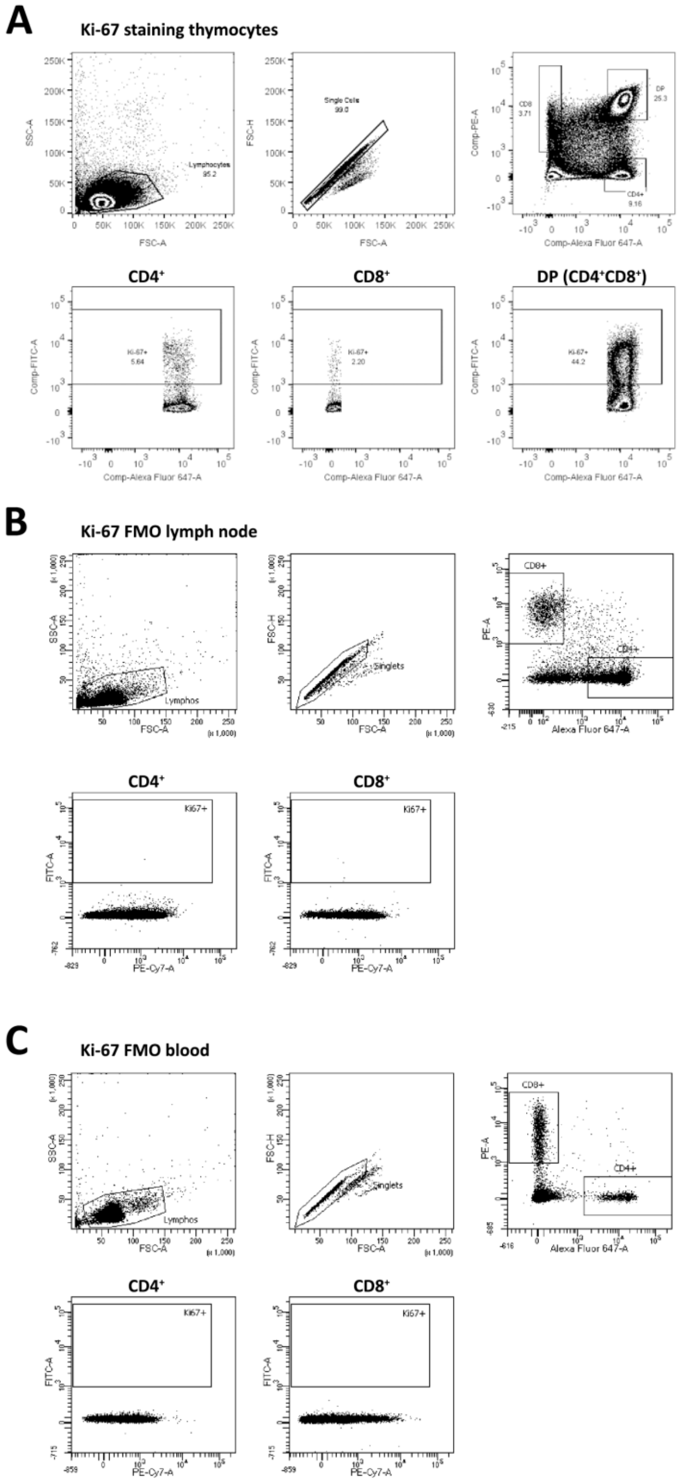

Sup. Figure 5. Positive and negative controls for intracellular Ki-67 staining. (A) Intracellular Ki-67 staining of $CD4^+$, $CD8^+$ and double positive ($CD4^+CD8^+$) thymocytes of a representative goat. (B and C) Ki-67 fluorescence minus one (FMO) staining of $CD4^+$, $CD8^+$ cells from LN and blood of a representative goat.

Sup. Table 1. Estimates for deuterium enrichment in plasma and granulocytes.

	Parameter	Estimate (95% confidence limits)
**Plasma**	δ	0.08433 (0.07486;0.09649)
	f	0.03381 (0.03184;0.03501)
**Granulocytes**	c	4.42 (4.39;4.66)

Sup. Table 2. Average turnover rate per day for $CD4^+$ and $CD8^+$ T-cells from blood, BM and LN.

	Average turnover rate (95% confidence limits)			
	$CD4^+CCR7^-$ (memory)	$CD4^+CCR7^+$ (naive)	$CD8^+CCR7^-$ (memory)	$CD8^+CCR7^+$ (naive)
**Blood**	0.0227 (0.0129;0.0374)	0.0047 (0.0019;0.0146)	0.0308 (0.0173;0.2008)	0.0005 (0.0003;0.0121)
**BM**	0.0199 (0.011;0.0466)	ND	0.0184 (0.0104;0.1340)	ND
**LN**	0.0186 (0.0102;0.0359)	0.0012 (0.0009;0.0245)	0.0074 (0.0054; 0.0582)	0.0007 (0.0004;0.0106)

ND: not determined

Sup. Table 3. Differentially expressed genes

$CD4^+$ T-cells Memory vs Naive			
Gene Name	Log fold change	adj. P value	Literature
ADARB1	-2.54915	0.002391	(Grigoryev et al., 2011)
ADARB1	-2.46267	0.003752	
BACH2	-1.87584	0.008572	(Hu & Chen, 2013a)
BACH2	-1.56094	0.041767	
BMP4	-1.10496	0.016446	(Martínez et al., 2015)
CADM1	-2.9022	0.007051	(Yeh, Sidhu, & Chan, 2008a)
CADM1	-2.88951	0.002837	
CADM1	-2.82384	0.004798	
CADM1	-2.77005	0.006469	
CADM1	-2.67598	0.006251	
CADM1	-2.67318	0.005128	
CCR4	1.991457	0.011533	(Marshall et al., 2011a)
CCR7	-2.40471	0.00941	(Marshall et al., 2011b; Sallusto, Geginat, & Lanzavecchia, 2004; Sallusto, Lenig, Förster, Lipp, & Lanzavecchia, 1999)
CHST11	1.943514	0.031714	(Hu & Chen, 2013b)
CRLF2	1.39184	0.043779	(Mitchell et al., 2015)
DLG4	-1.57907	0.014365	(Ludford-Menting et al., 2005)
DLG4	-1.40352	0.048212	
ERN1	1.433865	0.007051	(Brinza et al., 2016a)
FLNB	-1.43322	0.034901	(Ishihara et al., 2015)
FLNB	-1.40762	0.021134	
FLNB	-1.36113	0.034838	
FLNB	-1.30816	0.027905	
GHR	1.348845	0.000174	(Spadaro et al., 2016)

GHR	1.387197	0.000179	
GHR	1.424572	0.000174	
GHR	1.451081	0.00044	
GHR	1.670101	0.000159	
HNRNPLL	1.1555	0.041836	(Zuopeng Wu et al., 2008)
HNRNPLL	1.330412	0.010945	
IL17RB	1.825036	0.003655	(Lavorgna, Matsuoka, & Harhaj, 2014; Wang et al., 2006)
IL17RB	1.967296	0.003752	
IL4	1.788038	0.00938	(Romagnani, 1992)
OTUD7A	-1.57246	0.00022	(Zuopeng Wu et al., 2008)
PDK1	-1.86132	0.040798	(Park et al., 2009)
PELI3	-1.3658	0.008258	(W. Jin, Chang, & Sun, 2012)
PELI3	-1.00036	0.037282	
PLD2	1.443367	0.007889	(Chandrasekaran, 2010)
PTPN13	1.222311	0.048712	(Stanford, Rapini, & Bottini, 2012)
PTPN13	1.228839	0.03175	
PTPN13	1.269886	0.015939	
PTPN13	1.273159	0.038463	
PTPN13	1.404583	0.008258	
PTPN13	1.417017	0.039764	
PTPN13	1.428352	0.048029	
RUNX2	2.464744	0.019147	(Hu & Chen, 2013b)
RUNX2	2.478845	0.03852	
RUNX2	2.547863	0.021134	
S100A11	1.207338	0.048574	(Haining et al., 2008)
SELL	-2.83203	0.006251	(Sallusto et al., 2004, 1999)
SOX4	-2.04995	0.046482	(Hu & Chen, 2013b)
TRPC3	-1.69726	0.001252	(Wenning et al., 2011)
ZBTB16	1.739579	0.048029	(Zhang, Laouar, Denzin, & Sant'Angelo, 2015)
ZBTB16	1.965289	0.021788	
ZBTB44	-1.02269	0.041767	(Bhattacharyya et al., 2017)
**CD8^{+} T-cells Memory vs Naive**			
Gene Name	Log fold change	adj. P value	Literature
ACTN1	-2.02701	0.004779	(Lee, Hanspers, Barker, Korn, & McCune, 2004)
ACTN1	-2.01951	0.006649	
ACTN1	-1.98664	0.006654	
ADA	-1.44861	0.04267	(Martinez-Navio et al., 2011)
ADA	-1.38588	0.047133	
ADRB2	2.03203	0.020589	(Slota, Shi, Chen, Bevans, & Weng, 2015)
ANXA6	-1.47009	0.024847	(Wong, Chen, Wu, Liu, & Zheng, 2015)
ANXA6	-1.62705	0.038457	
ATP1B3	1.17328	0.019913	(Arsenio et al., 2014)
BACH2	-1.78332	0.00667	(Hu & Chen, 2013b)
BACH2	-1.73338	0.011494	
BCL2L11	1.848809	0.017825	(Wojciechowski et al., 2007)
BCL2L11	1.740505	0.027247	
BCL2L11	1.730477	0.028292	
BCL2L11	1.678561	0.030738	
BCL2L11	1.678507	0.032155	
CADM1	-2.3757	0.006235	(Yeh, Sidhu, & Chan, 2008b)
CADM1	-2.34733	0.006582	
CADM1	-2.49651	0.006754	
CADM1	-2.63067	0.007128	
CADM1	-2.37436	0.007607	
CADM1	-2.29788	0.008732	
CAMK2G	1.0616	0.003486	(Lin et al., 2005)
CAMK2G	1.066896	0.004779	
CAMK2G	1.031444	0.005023	
CAMK2G	1.039734	0.005316	
CAMK2G	1.048958	0.006235	
CAMK2G	1.093162	0.006939	
CAMK2G	1.07089	0.008791	
CAMK2G	1.027993	0.008791	
CAMK2G	1.005068	0.010375	
CCR7	-3.89792	0.000567	(Sallusto et al., 2004, 1999)

CD244	2.277336	0.020215	(Weng, Araki, & Subedi, 2012)
CD244	2.277336	0.020215	
CD2AP	-1.25602	0.045375	(Bachmann, Barner, & Kopf, 1999)
CD2AP	-1.44232	0.046136	
CD58	0.746696	0.029956	(Weng et al., 2012)
CRACR2A	1.459777	0.048532	(Srikanth et al., 2017)
CTLA4	-2.31751	0.031419	(Jago, Yates, Saraiva Câmara, Lechler, & Lombardi, 2004)
EFHD2	1.436447	0.006198	(Haining et al., 2008)
FOSL2	1.658675	0.03285	(Hu & Chen, 2013b)
GZMA	2.722409	0.022504	(Haining et al., 2008)
GZMA	2.722409	0.022504	
HDAC7	-1.27734	0.008732	(Myers et al., 2017)
HDAC7	-1.29071	0.009377	
HDAC7	-1.2653	0.011898	
HDAC7	-1.29661	0.012247	
HDAC7	-1.2668	0.012397	
HDAC7	-1.23558	0.012807	
HDAC7	-1.24396	0.017282	
IFNAR1	-1.08905	0.044404	(Arsenio et al., 2014)
IL12RB2	2.074168	0.031711	(Cleary et al., 2003)
IL21R	-1.70319	0.030678	(Zheng Wu et al., 2005)
IL21R	-1.69447	0.034067	
IL6ST	-2.01471	0.029956	(Marshall et al., 2011b)
IL7R	-1.49989	0.034417	(Arsenio et al., 2014; Holmes, He, Xu, & Lee, 2005)
ITGA1	-1.54763	0.032364	(Brinza et al., 2016b)
ITGAL	1.643297	0.042275	(Wong et al., 2015)
ITGAL	1.723651	0.045776	
ITGB2	1.439598	0.021548	(Wong et al., 2015)
ITGB2	1.421697	0.023959	
LEF1	-3.80763	0.001203	(Willinger et al., 2006)
LEF1	-3.83547	0.001302	
LEF1	-3.91898	0.001697	
LEF1	-3.63355	0.002435	
MYC	-1.86997	0.012247	(Wong et al., 2015)
MYC	-1.88266	0.012721	
NKG7	1.404262	0.025862	(Jenner et al., 2009)
OTUD7A	-1.42095	0.000921	(Zuopeng Wu et al., 2008)
PDK1	-3.33302	0.000682	(Park et al., 2009)
PTK2	-1.52327	0.005149	(Chapman & Houtman, 2014)
PTK2	-1.61764	0.007128	
PTK2	-1.6481	0.009241	
PTK2	-1.68791	0.009581	
PTK2	-1.62097	0.009793	
PTK2	-1.65695	0.010397	
PTK2	-1.59607	0.01093	
PTK2	-1.56553	0.011116	
PTK2	-1.71743	0.011116	
PTK2	-1.69744	0.011898	
PTK2	-1.49245	0.012666	
PTK2	-1.49781	0.012982	
PTK2	-1.58823	0.013563	
PTK2	-1.69485	0.014341	
PTK2	-1.3979	0.014885	
PTK2	-1.68131	0.017314	
PTK2	-1.51958	0.024539	
PTK2	-1.68575	0.025684	
PTK2	-1.5538	0.029796	
PTPRF	1.359712	0.043908	(Stanford et al., 2012)
RASGRP1	-1.5732	0.021548	(Ortega-Francisco et al., 2017)
RYR3	-1.36329	0.027199	(Thakur, Dadsetan, & Fomina, 2012)
SELL	-2.54407	0.006664	(Sallusto et al., 2004, 1999)
SEMA4D	-1.99565	0.006741	(Ortega-Francisco et al., 2017)
SEMA4D	-1.99418	0.007128	
SEMA4D	-1.98717	0.007764	
SH2D2A	1.96342	0.029379	(Sundvold et al., 2000)

SH2D2A	1.606576	0.040262	
SIDT1	-2.09569	0.000567	(Brinza et al., 2016b)
SIDT1	-2.06479	0.000768	
SPNS2	1.353609	0.023308	(Ortega-Francisco et al., 2017)
SPNS2	1.464658	0.027346	
TCF7	-1.93821	0.011123	(Willinger et al., 2006)
TCF7	-1.94776	0.01515	
TCF7	-1.80528	0.020589	
TCF7	-1.88955	0.022504	
TERT	-2.69408	0.023411	(Akbar & Vukmanovic-Stejic, 2007)
TGFBR3	-1.98545	0.012616	(Ortega-Francisco et al., 2017)
TGFBR3	-1.92901	0.013415	
TGFBR3	-1.91475	0.017404	
TIAM1	-3.6088	0.006198	(Gerard, van der Kammen, Janssen, Ellenbroek, & Collard, 2009)
TIAM1	-3.70851	0.006198	
TIAM1	-3.84106	0.006354	
TIAM1	-3.74174	0.006548	
TIAM1	-3.59794	0.009357	
TIAM1	-3.56771	0.010682	
TLR6	1.220077	0.012397	(B. Jin, Sun, Yu, Yang, & Yeo, 2012)
TNFRSF1B	1.653824	0.012724	(Weng et al., 2012)
TNFRSF1B	1.653824	0.012724	
TNFSF12	1.197349	0.008386	(Maecker et al., 2005)
TNFSF12	1.076618	0.025136	
TNFSF8	-2.3618	0.020589	(Nishimura et al., 2005)
UNC13D	1.396084	0.003248	(Cichocki et al., 2014)
UNC13D	1.292078	0.004564	
UNC13D	1.393111	0.006727	
**$CD8^+$ memory T-cells BM vs Blood**			
Gene Name	Log fold change	adj. P value	Literature
AIM1L	1.14699758	0.02676089	(Kumar et al., 2017)
ARHGAP18	1.09097448	0.04097176	(Kumar et al., 2017)
BACH2	1.51395879	0.01925728	(Hu & Chen, 2013b)
BACH2	1.32623424	0.04896827	
CCR9	2.62992229	0.00689682	(Cassani et al., 2011)
CD63	1.57277259	0.02149123	(Sincock, Mayrhofer, & Ashman, 1997)
CD63	1.6041947	0.01873893	
CD63	1.98179779	0.01986202	
CLCF1	1.16043424	0.03399945	(Kumar et al., 2017)
DYSF	1.667159	0.04809178	(Kumar et al., 2017)
DYSF	1.70676921	0.04946483	
DYSF	1.73281361	0.04731802	
DYSF	1.78459569	0.03399945	
DYSF	1.79339487	0.03552692	
DYSF	1.7994008	0.03033491	
DYSF	1.79995237	0.03924864	
DYSF	1.8006521	0.03592599	
DYSF	1.80346625	0.0289546	
DYSF	1.81189529	0.03399945	
DYSF	1.82852166	0.02721505	
DYSF	1.82914859	0.03513176	
DYSF	1.85341807	0.04465047	
DYSF	1.85905908	0.03694871	
DYSF	1.88247411	0.02676089	
DYSF	1.88277163	0.03112209	
DYSF	1.8866629	0.03140228	
FAM102A	1.03466669	0.04480112	(Kumar et al., 2017)
FAM129A	1.23756442	0.03880919	(Kumar et al., 2017)
FAM65B	1.41933439	0.03320681	(Kumar et al., 2017)
GPR25	2.71953101	0.01706119	(Kumar et al., 2017)
HIP1	1.26020867	0.01403986	(Kumar et al., 2017)
HIP1	1.26383191	0.01085178	
HIP1	1.28115137	0.00130614	
IFNGR1	1.23838062	0.04262082	(Kumar et al., 2017)
IFNGR1	1.2977877	0.03079968	

KLF2	1.39284657	0.02705716	(Kumar et al., 2017)
OCRL	1.38165747	0.02985422	(Kumar et al., 2017)
PPFIBP2	1.65867654	0.01757218	(Kumar et al., 2017)
RAP1GAP2	1.22722779	0.0449822	(Kumar et al., 2017)
RAP1GAP2	1.20028036	0.04866786	
RASA3	1.60345786	0.00524516	(Kumar et al., 2017)
RASA3	1.56944764	0.0086063	
RASA3	1.53848845	0.0091894	
RASA3	1.52353461	0.00874084	
RASGRP2	1.13014705	0.01312975	(Kumar et al., 2017)
RASSF3	1.27620496	0.01536454	(Kumar et al., 2017)
RASSF4	1.19424201	0.00474526	
RASSF4	1.24669139	0.00471071	
RASSF4	1.35343455	0.00524516	
RASSF4	1.41585924	0.00204417	
S1PR1	1.91192176	0.04618508	(Okhrimenko et al., 2014)
	1.86101968	0.04950476	
SLCO3A1	2.01749058	0.03140228	(Kumar et al., 2017)
SLCO3A1	1.68061642	0.00212074	
SPHK1	1.63389017	0.00267473	(Yamanaka et al., 2004)
SPHK1	1.71277563	0.00202112	
SPHK1	1.7733981	0.00042174	
SPHK1	1.87209887	0.00128658	
SPHK1	1.87624265	0.00078737	
SSBP3	1.42170563	0.00871699	(Kumar et al., 2017)
SSBP3	1.40698106	0.00434456	
TGFBI	2.4996392	0.01862216	(Kumar et al., 2017)

Akbar, A. N., and M. Vukmanovic-Stejic. 2007. "Telomerase in T Lymphocytes: Use It and Lose It?" *The Journal of Immunology* 178(11): 6689–94.

Arsenio, Janilyn et al. 2014. "Early Specification of CD8+ T Lymphocyte Fates during Adaptive Immunity Revealed by Single-Cell Gene-Expression Analyses." *Nature Immunology* 15(4): 365–72.

Bachmann, M F, M Barner, and M Kopf. 1999. "CD2 Sets Quantitative Thresholds in T Cell Activation." *The Journal of experimental medicine* 190(10): 1383–92.

Bhattacharyya, Mitra et al. 2017. "Regulation of CD4 T Cells and Their Effects on Immunopathological Inflammation Following Viral Infection." *Immunology* 152(2): 328–43.

Brinza, Lilia et al. 2016a. "Immune Signatures of Protective Spleen Memory CD8 T Cells." *Scientific Reports* 6(July): 1–12.

Cassani, Barbara et al. 2011. "Gut-Tropic T Cells That Express Integrin α4β7 and CCR9 Are Required for Induction of Oral Immune Tolerance in Mice." *Gastroenterology* 141(6): 2109–18.

Chandrasekaran, Uma. 2010. "Phospholipase D Signaling in T Cells."

Chapman, Nicole M., and Jon C. D. Houtman. 2014. "Functions of the FAK Family Kinases in T Cells: Beyond Actin Cytoskeletal Rearrangement." *Immunologic Research* 59(1–3): 23–34.

Cichocki, Frank et al. 2014. "Transcriptional Regulation of Munc13-4 Expression in Cytotoxic Lymphocytes Is Disrupted by an Intronic Mutation Associated with a Primary Immunodeficiency." *The Journal of Experimental Medicine* 211(6): 1079–91.

Cleary, A. M. et al. 2003. "Impaired Accumulation and Function of Memory CD4 T Cells in Human IL-12 Receptor 1 Deficiency." *The Journal of Immunology* 170(1): 597–603.

Gerard, A. et al. 2009. "The Rac Activator Tiam1 Controls Efficient T-Cell Trafficking and Route of Transendothelial Migration." *Blood* 113(24): 6138–47.

Grigoryev, Y. A. et al. 2011. "MicroRNA Regulation of Molecular Networks Mapped by Global MicroRNA, mRNA, and Protein Expression in Activated T Lymphocytes." *The Journal of Immunology* 187(5): 2233–43.

Haining, W. N. et al. 2008. "Identification of an Evolutionarily Conserved Transcriptional Signature of CD8 Memory Differentiation That Is Shared by T and B Cells." *The Journal of Immunology* 181(3): 1859–68.

Holmes, S., M. He, T. Xu, and P. P. Lee. 2005. "Memory T Cells Have Gene Expression Patterns Intermediate between Naive and Effector." *Proceedings of the National Academy of Sciences* 102(15): 5519–23. Hu, Guangan, and Jianzhu Chen. 2013a. "A Genome-Wide Regulatory Network Identifies Key Transcription Factors for Memory CD8+ T-Cell Development." *Nature Communications* 4(7): 1400–1414.

Ishihara, Sayaka et al. 2015. "Dual Functions of Rap1 Are Crucial for T-Cell Homeostasis and Prevention of Spontaneous Colitis." *Nature Communications* 6: 1–16.

Jago, C. B. et al. 2004. "Differential Expression of CTLA-4 among T Cell Subsets." *Clinical and Experimental Immunology* 136(3): 463–71.

Jenner, R. G. et al. 2009. "The Transcription Factors T-Bet and GATA-3 Control Alternative Pathways of T-Cell Differentiation through a Shared Set of Target Genes." *Proceedings of the National Academy of Sciences* 106(42): 17876–81.

Jin, Bo et al. 2012. "The Effects of TLR Activation on T-Cell Development and Differentiation." *Clinical and Developmental Immunology* 2012(Figure 1).

Jin, Wei, Mikyoung Chang, and Shao Cong Sun. 2012. "Peli: A Family of Signal-Responsive E3 Ubiquitin Ligases Mediating TLR Signaling and T-Cell Tolerance." *Cellular and Molecular Immunology* 9(2): 113–22.

Kumar, Brahma V. et al. 2017. "Human Tissue-Resident Memory T Cells Are Defined by Core Transcriptional and Functional Signatures in Lymphoid and Mucosal Sites." *Cell Reports* 20(12): 2921–34.

Lavorgna, Alfonso, Masao Matsuoka, and Edward William Harhaj. 2014. "A Critical Role for IL-17RB Signaling in HTLV-1 Tax-Induced NF-κB Activation and T-Cell Transformation." *PLoS Pathogens* 10(10): 1–16.
Lee, Myeong Sup et al. 2004. "Gene Expression Profiles during Human CD4+ T Cell Differentiation." *International Immunology* 16(8): 1109–24.
Lin, Meei Yun et al. 2005. "A Pivotal Role for the Multifunctional Calcium/ Calmodulin-Dependent Protein Kinase II in T Cells: From Activation to Unresponsiveness." *The Journal of Immunology*: 5583–92.
Ludford-Menting, Mandy J. et al. 2005. "A Network of PDZ-Containing Proteins Regulates T Cell Polarity and Morphology during Migration and Immunological Synapse Formation." *Immunity* 22(6): 737–48.
Maecker, Heather et al. 2005. "TWEAK Attenuates the Transition from Innate to Adaptive Immunity." *Cell* 123(5): 931–44.
Marshall, Heather D. et al. 2011a. "Differential Expression of Ly6C and T-Bet Distinguish Effector and Memory Th1 CD4+Cell Properties during Viral Infection." *Immunity* 35(4): 633–46.
Martinez-Navio, José M. et al. 2011. "Adenosine Deaminase Potentiates the Generation of Effector, Memory, and Regulatory CD4 + T Cells." *Journal of Leukocyte Biology* 89(1): 127–36.
Martínez, Víctor G. et al. 2015. "The BMP Pathway Participates in Human Naive $CD4^{+}$ T Cell Activation and Homeostasis." *PLoS ONE* 10(6): 1–18.
Mitchell, Christopher J. et al. 2015. "A Multi-Omic Analysis of Human Naï¿½ve CD4+ T Cells." *BMC Systems Biology* 9(1): 1–16.
Myers, Darienne R. et al. 2017. "Tonic LAT-HDAC7 Signals Sustain Nur77 and Irf4 Expression to Tune Naive CD4 T Cells." *Cell Reports* 19(8): 1558–71.
Nishimura, Hitoshi et al. 2005. "A Novel Role of CD30/CD30 Ligand Signaling in the Generation of Long-Lived Memory CD8+ T Cells." *Journal of immunology (Baltimore, Md. : 1950)* 175(7): 4627–34.
Okhrimenko, Anna et al. 2014. "Human Memory T Cells from the Bone Marrow Are Resting and Maintain Long-Lasting Systemic Memory." *Proceedings of the National Academy of Sciences of the United States of America* 111(25): 9229–34.
Ortega-Francisco, Sandra et al. 2017. "TβRIII Is Induced by TCR Signaling and Downregulated in FoxP3 + Regulatory T Cells." *Biochemical and Biophysical Research Communications* 494(1–2): 82–87.
Park, Sung-gyoo et al. 2009. "Phosphoinositide-Dependent Kinase 1 Integrates T Cell Receptor and CD28 Co-Receptor Signaling to Effect NF-κB Induction and T Cell Activation." *Nature immunology* 10(2): 158–66.
Romagnani, S. 1992. "Human TH1 and TH2 Subsets: Regulation of Differentiation and Role in Protection and Immunopathology." *International Archives of Allergy and Immunology* 98(4): 279–85.
Sallusto, Federica et al. 1999. "Two Subsets of Memory T Lymphocytes with Distinct Homing Potentials and Effector Functions." *Nature* 401(6754): 708–12.
Sallusto, Federica, Jens Geginat, and Antonio Lanzavecchia. 2004. "Central Memory and Effector Memory T Cell Subsets: Function, Generation, and Maintenance." *Annual review of immunology* 22(1): 745–63.
Sincock, Paul M., Graham Mayrhofer, and Leonie K. Ashman. 1997. "Localization of the Transmembrane 4 Superfamily (TM4SF) Member PETA-3 (CD151) in Normal Human Tissues: Comparison with CD9, CD63, and α5β1 Integrin." *Journal of Histochemistry & Cytochemistry* 45(4): 515–25.
Slota, Christina et al. 2015. "Norepinephrine Preferentially Modulates Memory CD8 T Cell Function Inducing Inflammatory Cytokine Production and Reducing Proliferation in Response to Activation." *Brain, Behavior, and Immunity* 46(410): 168–79.
Spadaro, Olga et al. 2016. "Growth Hormone Receptor Deficiency Protects against Age-Related NLRP3 Inflammasome Activation and Immune Senescence." *Cell Reports* 14(7): 1571–80.
Srikanth, Sonal et al. 2017. "HHS Public Access." 9(420): 1–28. https://www.ncbi.nlm.nih.gov/pmc/articles/PMC5013727/pdf/nihms812852.pdf.
Stanford, Stephanie M., Novella Rapini, and Nunzio Bottini. 2012. "Regulation of TCR Signalling by Tyrosine Phosphatases: From Immune Homeostasis to Autoimmunity." *Immunology* 137(1): 1–19.
Sundvold, V et al. 2000. "T Cell-Specific Adapter Protein Inhibits T Cell Activation by Modulating Lck Activity." *Journal of immunology (Baltimore, Md.: 1950)* 165(6): 2927–31.
Thakur, Pratima, Sepehr Dadsetan, and Alla F. Fomina. 2012. "Bidirectional Coupling between Ryanodine Receptors and Ca2+ Release-Activated Ca2+ (CRAC) Channel Machinery Sustains Store-Operated Ca2+ Entry in Human T Lymphocytes." *Journal of Biological Chemistry* 287(44): 37233–44.
Uhlen, M. et al. 2015. "Tissue-Based Map of the Human Proteome." *Science* 347(6220): 1260419–1260419.
Wang, Yui Hsi et al. 2006. "Maintenance and Polarization of Human TH2 Central Memory T Cells by Thymic Stromal Lymphopoietin-Activated Dendritic Cells." *Immunity* 24(6): 827–38. Weng, Nan-ping, Yasuto Araki, and Kalpana Subedi. 2012. "The Molecular Basis of the Memory T Cell Response: Differential Gene Expression and Its Epigenetic Regulation." *Nature Reviews Immunology* 12(4): 306–15.
Wenning, Anna S. et al. 2011. "TRP Expression Pattern and the Functional Importance of TRPC3 in Primary Human T-Cells." *Biochimica et Biophysica Acta - Molecular Cell Research* 1813(3): 412–23.
Willinger, T. et al. 2006. "Human Naive CD8 T Cells Down-Regulate Expression of the WNT Pathway Transcription Factors Lymphoid Enhancer Binding Factor 1 and Transcription Factor 7 (T Cell Factor-1) Following Antigen Encounter In Vitro and In Vivo." *The Journal of Immunology* 176(3): 1439–46.
Wojciechowski, Sara et al. 2007. "Bim/Bcl-2 Balance Is Critical for Maintaining Naive and Memory T Cell Homeostasis." *The Journal of Experimental Medicine* 204(7): 1665–75.
Wong, Chunshu et al. 2015. "A Critical Role for the Regulated Wnt–Myc Pathway in Naive T Cell Survival." *The Journal of Immunology* 194(1): 158–67.
Wu, Zuopeng et al. 2008. "Memory T Cell RNA Rearrangement Programmed by Heterogeneous Nuclear Ribonucleoprotein hnRNPLL." *Immunity* 29(6): 863–75. Yamanaka, Masayoshi et al. 2004. "Sphingosine Kinase 1 (SPHK1) Is Induced by Transforming Growth Factor-β and Mediates TIMP-1 up-Regulation." *Journal of Biological Chemistry* 279(52): 53994–1.
Yeh, Jung Hua, Sachdev S. Sidhu, and Andrew C. Chan. 2008a. "Regulation of a Late Phase of T Cell Polarity and Effector Functions by Crtam." *Cell* 132(5): 846–59.
Zhang, Sai, Amale Laouar, Lisa K. Denzin, and Derek B. Sant'Angelo. 2015. "Zbtb16 (PLZF) Is Stably Suppressed and Not Inducible in Non-Innate T Cells via T Cell Receptor-Mediated Signaling." *Scientific Reports* 5(July): 1–13.
